# Diagnosis and Management of Lean Metabolic Dysfunction-Associated Steatotic Liver Disease (MASLD): A Systematic Review

**DOI:** 10.7759/cureus.71451

**Published:** 2024-10-14

**Authors:** Basile Njei, Prince Ameyaw, Yazan Al-Ajlouni, Lea-Pearl Njei, Sarpong Boateng

**Affiliations:** 1 Department of Medicine, Yale School of Medicine, New Haven, USA; 2 Department of Internal Medicine, Bridgeport Hospital, Yale New Haven Health, Bridgeport, USA; 3 Department of Medicine, New York Medical College, New York, USA; 4 Department of Medicine, University of Maryland, Baltimore, USA; 5 Department of Medicine, Yale Affiliated Hospitals Program, New Haven, USA

**Keywords:** diagnosis, lean masld, lean phenotype management, metabolic dysfunction-associated steatotic liver disease, nonobese fatty liver disease

## Abstract

Lean metabolic dysfunction-associated steatotic liver disease (MASLD) defies traditional views of fatty liver diseases by manifesting in nonobese individuals. The renaming from nonalcoholic fatty liver disease to MASLD underscores a broader understanding of its pathophysiology, highlighting the complex interplay of metabolic factors beyond obesity. Despite its clinical importance, diagnosing and managing lean MASLD remains challenging due to its historical ties to obesity and a general lack of awareness about its unique characteristics.

On December 4, 2023, a systematic literature search was conducted across six databases, focusing on peer-reviewed studies in English related to the diagnosis and management of lean MASLD. This study was registered with the International Prospective Register of Systematic Reviews (CRD42023489308). Out of 95 studies following Preferred Reporting Items for Systematic Reviews and Meta-Analyses guidelines, 43 addressed diagnosis and surveillance, whereas 52 explored management strategies. The results revealed the difficulties in diagnosing lean MASLD, pointing out the limitations of traditional markers and the potential of advanced imaging techniques. Management strategies discussed included lifestyle changes and possible pharmacological treatments tailored to the specific metabolic features of this patient group.

The study highlights the necessity for increased clinical awareness, regular monitoring, and personalized therapeutic approaches for lean MASLD. It calls for further research to refine diagnostic criteria and develop targeted treatments, aiming to enhance care for individuals with lean MASLD.

## Introduction and background

Nonalcoholic fatty liver disease (NAFLD) refers to a spectrum of chronic liver disease defined by ≥5% of hepatic steatosis ranging from the absence (nonalcoholic fatty liver) and presence of hepatocellular injury with or without fibrosis (nonalcoholic steatohepatitis, NASH) to cirrhosis and hepatocellular carcinoma (HCC) in the absence of secondary causes for having hepatic steatosis [1,2]. The absence of significant alcohol use and other secondary etiologies of hepatic steatosis was required for diagnosis [1]. The term metabolic dysfunction-associated steatotic liver disease (MASLD) was introduced by an international consensus group to replace NAFLD due to concerns of stigmatization and inadequate description of the disease etiology by the terms "nonalcoholic" and "fatty" [3]. MASLD highlights the coexistence and synergistic roles of the individual components of metabolic syndrome and alcohol use [4], thereby affecting policy change geared toward enhancing case identification and management [3]. While metabolic syndrome and obesity have been strongly linked with MASLD, a subset of patients termed “lean MASLD” exhibit hepatic steatosis without being overweight or obese [5]. A recent meta-analysis by Riazi et al. reported the overall global prevalence of MASLD in adults as 32.4% (95% confidence interval, CI, 29.9-34.9) [6], representing an alarming increase from previously reported figures (e.g., 25.24%-29.8%) [7,8]. About 7%-20% of the population with MASLD are lean or nonobese as defined by the WHO cutoff (body mass index, BMI, <25 kg/m^2^ in Asia and <30 kg/m^2^ in the rest of the world) [2,9,10]. A systematic review and meta-analysis by Ye et al. involving 93 studies from 24 countries showed a prevalence of 19.2% and 40.8% for lean and nonobese patients, respectively, in the MASLD population [11]. The prevalence of nonobese MASLD in the general population varied from 25% or lower in some countries (e.g., Malaysia and Pakistan) to higher than 50% in others (e.g., Austria, Mexico, and Sweden) [11].

Lean MASLD can be categorized into subtypes: the more prevalent subtype involving “nonobese patients who may be overweight (BMI 88th-95th percentile for age) and/or have increased waist circumference and visceral adipose tissue” and the less common subtype of lean subjects without excess adipose tissue [12]. The second group may develop fatty liver from secondary etiologies such as protein-energy malnutrition, medication adverse effects, and inflammatory, metabolic, and genetically predisposing conditions [12,13].

Despite its widespread use, BMI has its inadequacies in accounting for body fat composition, especially visceral adiposity [14]. Kim et al. demonstrated the superior association of visceral fat obesity with MASLD in nonobese subjects compared to BMI [15]. Other studies have shown similar findings of abdominal obesity, waist-to-hip ratio, muscle fat content, and sarcopenia being strong predictors of MASLD development, severity, and outcomes [16-19]. Further research is needed to explore these factors in nonobese patients better. Lean MASLD has been associated with a favorable profile of metabolic comorbidities and progression to cirrhosis [5,9]. However, they experience similar risks of hepatic decompensation, malignancy, and cardiovascular diseases than their obese counterparts [5]. There are emerging data that patients with lean MASLD have higher rates of advanced liver fibrosis-related and all-cause mortality compared to nonlean MASLD patients [5,13,20,21]. All-cause mortality, cardiovascular mortality, and long-term outcomes are worse in patients with lean MASLD compared to lean, healthy patients without MASLD, suggesting other risk-contributing factors beyond BMI [9,22]. Recognizing and managing lean MASLD is crucial due to its potential complications and distinct clinical characteristics [23]. This review provides insights into its diagnosis and management, emphasizing the unique challenges and considerations it presents.

## Review

Methods

A systematic literature search was conducted following the Preferred Reporting Items for Systematic Reviews and Meta-Analyses (PRISMA) guidelines to comprehensively review the existing literature on diagnosing and managing lean MASLD. The search was performed on December 4, 2023. The protocol for this systematic review is registered with the International Prospective Register of Systematic Reviews (CRD42023489308). This article was previously posted to the Research Square preprint server on February 15, 2024.

Search Strategy and Selection Criteria

A set of keywords specific to the research question was developed and employed, utilizing Boolean operators to enhance search efficiency. The search covered multiple databases: 1) PubMed, 2) MEDLINE, 3) PsycINFO, 4) Web of Science, 5) Excerpta Medica dataBASE (EMBASE), 6) ScienceDirect, and 7) Cumulative Index to Nursing and Allied Health Literature (CINAHL). The detailed search terms are provided in Appendix 1.

**Figure 1:**
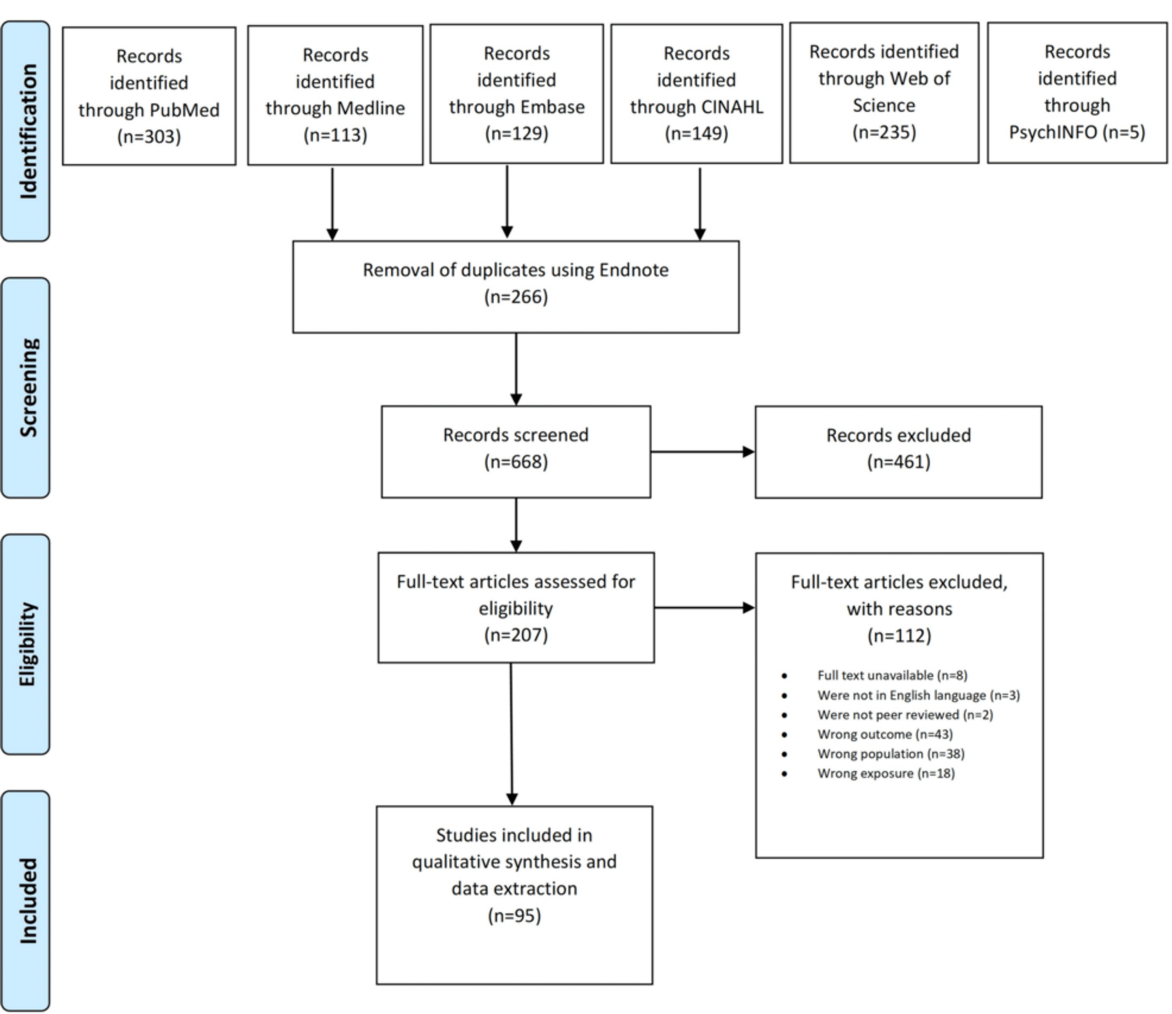
PRISMA study selection flow diagram outlining the literature review process when searching for articles on various databases CINAHL: Cumulative Index to Nursing and Allied Health Literature; PRISMA: Preferred Reporting Items for Systematic Reviews and Meta-Analyses

As shown in the PRISMA flow diagram in Figure 1, after the initial retrieval of articles (n = 934), duplicates were removed using the Endnote reference manager software (Clarivate, Philadelphia, PA), resulting in 668 unique articles. Two independent investigators (B.N. and Y.A.A.) evaluated titles and abstracts, leading to the exclusion of irrelevant articles (n = 461). Full-text versions of the remaining articles were obtained and assessed for eligibility based on predefined inclusion and exclusion criteria.

Inclusion and Exclusion Criteria

For inclusion in the review, studies had to meet specific criteria to address the research question comprehensively. The eligibility criteria included the following requirements: 1) the studies must have been conducted and published in the English language, 2) the studies should primarily report findings on the diagnosis and/or management of MASLD, 3) the target population should include lean individuals, 4) full-text papers must be available, and 5) the studies should have undergone peer review before final publication. The following criteria were used to exclude studies: 1) studies that did not undergo peer review, 2) studies with unavailable full-text papers, 3) studies written in languages other than English, and 4) studies designed as literature reviews, including scoping, systematic, narrative, or any other review format. The detailed criteria are outlined in Appendix 2, which provides a comprehensive guide for the screening process. Additionally, when a single study or dataset was documented in multiple publications, preference was given to the publication that conveyed outcomes and additional pertinent information aligned with the research query. Alternatively, consideration was given to the publication that was published in a more appropriate format, such as an original research article instead of a research letter.

Data Extraction and Presentation

A comprehensive approach to data collection and presentation was undertaken to synthesize information on diagnosing and managing lean MASLD among lean individuals. After the screening stage, a structured tabulated form was created for data extraction. The extraction process was conducted by a single reviewer using a predesigned table, which the coauthors critically reviewed. The data collection focused on various dimensions pertinent to each study, encompassing the name of the first author and the year of publication, study design, study location (country), population sociodemographic characteristics, diagnosis method or management approach, outcome investigated, and summary of the main findings.

The collected data were systematically organized within a tabular format without distinct categorization. This presentation offered a clear overview of the studied population, emphasizing diagnostic and management aspects. The textual format was employed not only to identify primary determinants documented in the existing literature but also to highlight gaps in the current body of knowledge pertaining to the diagnosis and management of lean MASLD among lean individuals. Given the heterogeneity in the topic and the diverse domains explored, we opted for a narrative synthesis of results. This integrated approach to data extraction and presentation ensured a comprehensive synthesis of information, providing a thorough understanding of the current research landscape on diagnosing and managing MASLD in lean populations. Through this method, areas requiring further investigation were highlighted, contributing to the ongoing exploration of effective strategies for diagnosing and managing MASLD in lean individuals.

Quality Assessment

The quality assessment of included randomized controlled trials (RCTs) was performed using the revised Cochrane risk of bias tool (RoB2), developed by the Cochrane Collaboration (United Kingdom) and modified to ensure standardized scoring [24]. This tool is recognized for its comprehensive evaluation of key domains, ensuring a robust assessment of each study's methodological quality.

Two independent reviewers conducted the quality assessment, with any discrepancies resolved through discussion and consensus. The RoB2 tool was applied to appraise the risk of bias in the following domains: randomization process, deviations from intended interventions, missing outcome data, measurement of the outcome, and selection of the reported result. Each domain was evaluated for low, some concerns, or high risk of bias, providing a nuanced understanding of the overall study quality.

Results

Literature Search Results

The systematic literature search yielded a total of 934 studies. These studies were downloaded from various databases, including 303 from PubMed, 113 from Medline, 129 from EMBASE, 149 from CINAHL, 235 from Web of Science, and five from PsycINFO. Using Endnote, a software for reference management, duplicates (n = 266) were removed. Six hundred sixty-eight unique articles were screened based on title and abstract. At this stage, a total of 461 studies were excluded due to irrelevant outcome (n = 169), irrelevant population (n = 164), irrelevant exposure (n = 115), and irrelevant study design (n = 13). Full-text versions of the remaining 207 articles were sought, and 199 full-text studies were reviewed, with eight studies excluded due to the unavailability of full-text versions. After the full-text review, an additional 104 studies were excluded. The reasons for exclusion at this stage were as follows: irrelevant outcome (n = 43), irrelevant population (n = 38), irrelevant exposure (n = 18), non-English language (n = 3), and non-peer reviewed (n = 2). Consequently, 95 studies were included in the final synthesis.

Study Characteristics

A total of 95 studies were included in this review. Forty-three studies focused on the diagnosis of lean MASLD. Among those studies, the number of participants ranged from 130 individuals [25] to 2,715 individuals [26]. Studies focused on diagnoses were conducted in various geographical contexts, including 13 in the United States, four in China, and three in France. Furthermore, those studies ranged in design, including literature reviews (n = 20), cohort studies (n = 11), and cross-sectional studies (n = 7). Similarly, in the studies focused on the management of MASLD (n = 52), the number of participants ranged from 31 individuals [27] to 11,593,409 individuals [28]. Moreover, 18 studies were conducted in the United States, followed by five studies in the United Kingdom, seven studies in Italy, and four studies in China. Literature reviews were the most prevalent among studies focused on MASLD management (n = 17). Other study designs included cohort studies (n = 10) and cross-sectional studies (n = 11).

Quality Assessment

**Figure 2:**
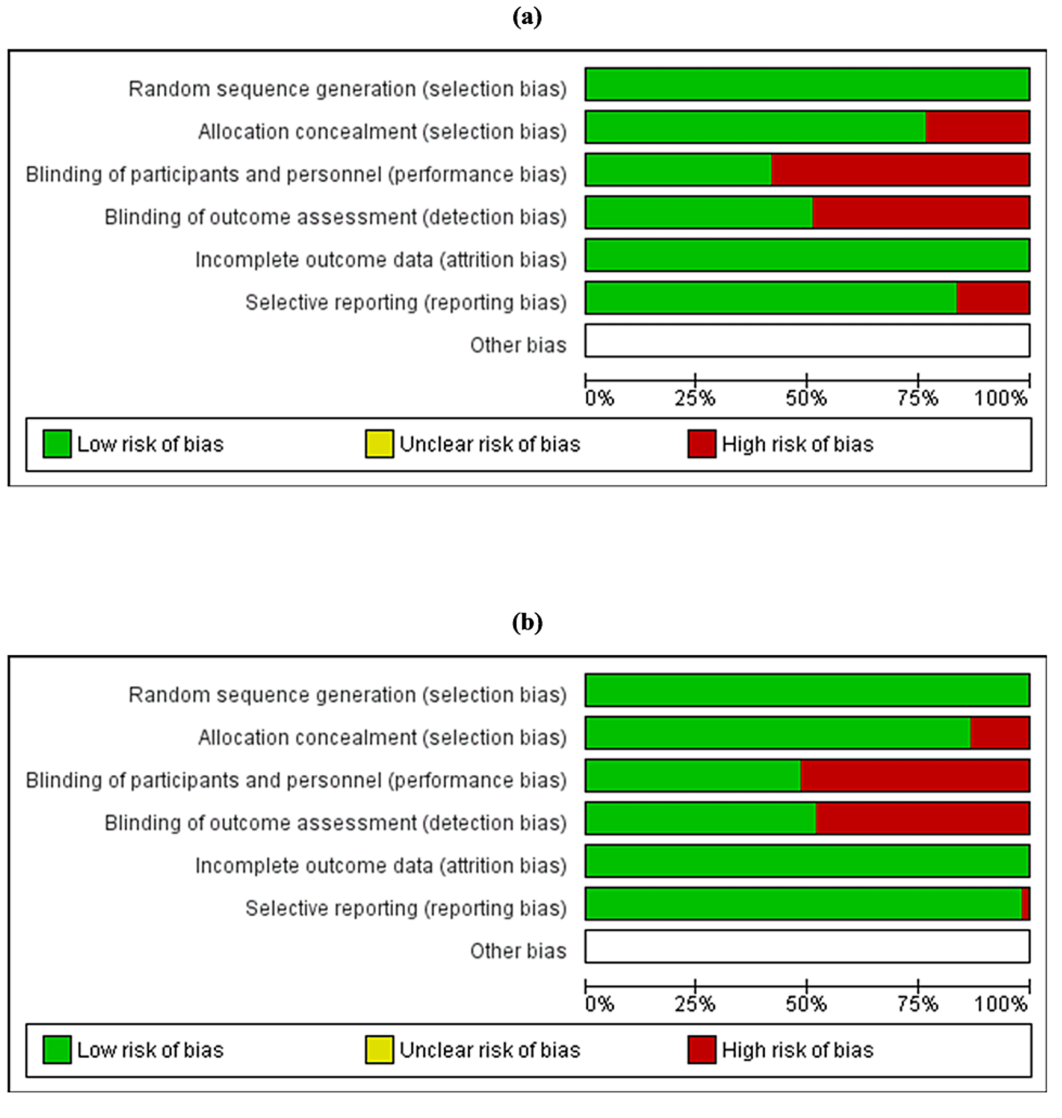
Summary of the results of quality assessment of included studies based on the RoB2 tool. Studies focusing on (a) MASLD diagnosis and (b) MASLD management MASLD: metabolic dysfunction-associated steatotic liver disease; RoB2: risk of bias 2

**Figure 3:**
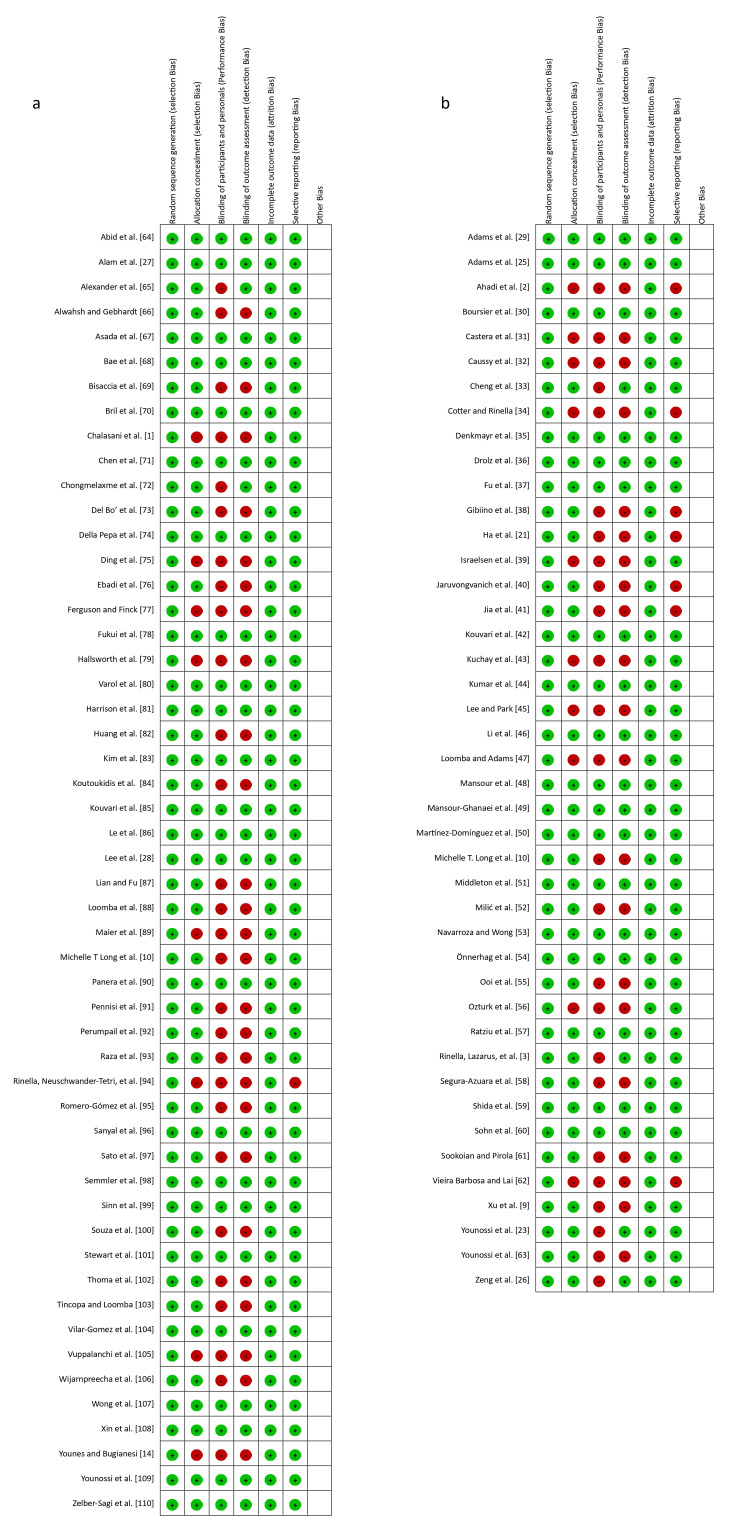
Overall risk of bias assessment in systematic review studies. Studies focusing on (a) MASLD diagnosis and (b) MASLD management MASLD: metabolic dysfunction-associated steatotic liver disease

In our quality assessment using the ROB2 tool, we evaluated 95 studies, with results depicted in Tables 1, 2 and Figures 2, 3. Most studies demonstrated a low risk of bias in random sequence generation, indicating proper randomization methods. However, allocation concealment showed notable high risk in some studies, suggesting issues in maintaining allocation sequence concealment. Performance bias, reflected in the blinding of participants and personnel, exhibited a substantially high risk of bias, highlighting inadequacies in blinding practices. Concerning the blinding of outcome assessment, detection bias also showed significant high risk, indicating challenges in maintaining blinded assessments. In contrast, most studies had a low risk of bias for incomplete outcome data, suggesting appropriate handling of missing data. Selective reporting was mostly at low risk, though a few studies still exhibited high risk, indicating potential reporting biases. Other biases were not prominently marked, suggesting minimal or unassessed biases in these categories.

**Table 1:** ROB analysis of MASLD diagnostic methods ROB: risk of bias; MASLD: metabolic dysfunction-associated steatotic liver disease

Study	ROB: sequence generation	ROB: allocation concealment	ROB: blinding of personnel/patient	ROB: blinding of outcome assessor	ROB: incomplete outcome data	ROB: selective outcome reporting
Adams et al. [29]	Low bias	Low bias	Low bias	Probably low bias	Low bias	Probably low bias
Adams et al. [25]	Probably low bias	Low bias	Low bias	Low bias	Low bias	Low bias
Ahadi et al. [2]	Low bias	Probably high bias	Probably high bias	Probably high bias	Low bias	Probably high bias
Boursier et al. [30]	Probably low bias	Low bias	Low bias	Low bias	Low bias	Probably low bias
Castera et al. [31]	Low bias	Probably high bias	Probably high bias	Probably high bias	Low bias	Low bias
Caussy et al. [32]	Low bias	Probably high bias	Probably high bias	Probably high bias	Low bias	Low bias
Cheng et al. [33]	Low bias	Low bias	Probably high bias	Low bias	Low bias	Low bias
Cotter and Rinella [34]	Low bias	Probably high bias	Probably high bias	Probably high bias	Low bias	Probably high bias
Denkmayr et al. [35]	Probably low bias	Low bias	Low bias	Low bias	Low bias	Low bias
Drolz et al. [36]	Low bias	Low bias	Low bias	Probably low bias	Low bias	Probably low bias
Fu et al. [37]	Low bias	Low bias	Low bias	Low bias	Low bias	Low bias
Gibiino et al. [38]	Low bias	Low bias	Probably high bias	Probably high bias	Low bias	Probably high bias
Ha et al. [21]	Low bias	Low bias	Probably high bias	Probably high bias	Low bias	Probably high bias
Israelsen et al. [39]	Low bias	Probably high bias	Probably high bias	Probably high bias	Low bias	Low bias
Jaruvongvanich et al. [40]	Low bias	Low bias	Probably high bias	Probably high bias	Low bias	Probably high bias
Jia et al. [41]	Low bias	Low bias	Probably high bias	Probably high bias	Low bias	Probably high bias
Kouvari et al. [42]	Low bias	Low bias	Low bias	Probably low bias	Low bias	Probably low bias
Kuchay et al. [43]	Low bias	Probably high bias	Probably high bias	Probably high bias	Low bias	Low bias
Kumar et al. [44]	Low bias	Low bias	Low bias	Low bias	Low bias	Low bias
Lee and Park [45]	Low bias	Probably high bias	Probably high bias	Probably high bias	Low bias	Low bias
Li et al. [46]	Low bias	Low bias	Low bias	Probably low bias	Low bias	Low bias
Long et al. [10]	Low bias	Low bias	Probably high bias	Probably high bias	Low bias	Low bias
Loomba and Adams [47]	Low bias	Probably high bias	Probably high bias	Probably high bias	Low bias	Low bias
Mansour et al. [48]	Low bias	Probably low bias	Low bias	Low bias	Low bias	Probably low bias
Mansour-Ghanaei et al. [49]	Low bias	Low bias	Low bias	Low bias	Low bias	Low bias
Martínez-Domínguez et al. [50]	Low bias	Low bias	Low bias	Low bias	Probably low bias	Low bias
Middleton et al. [51]	Low bias	Low bias	Probably low bias	Probably low bias	Low bias	Low bias
Milić et al. [52]	Low bias	Low bias	Probably high bias	Probably high bias	Low bias	Low bias
Navarroza and Wong [53]	Low bias	Low bias	Low bias	Low bias	Probably low bias	Low bias
Önnerhag et al. [54]	Low bias	Low bias	Probably low bias	Low bias	Low bias	Low bias
Ooi et al. [55]	Low bias	Low bias	Probably high bias	Probably high bias	Low bias	Low bias
Ozturk et al. [56]	Low bias	Probably high bias	Probably high bias	Probably high bias	Low bias	Low bias
Ratziu et al. [57]	Low bias	Low bias	Probably low bias	Low bias	Low bias	Low bias
Rinella et al. [3]	Low bias	Low bias	Probably high bias	Low bias	Low bias	Low bias
Segura-Azuara et al. [58]	Low bias	Low bias	Probably high bias	Probably high bias	Low bias	Low bias
Shida et al. [59]	Low bias	Low bias	Low bias	Low bias	Probably low bias	Probably low bias
Sohn et al. [60]	Low bias	Low bias	Low bias	Low bias	Probably low bias	Low bias
Sookoian and Pirola [61]	Low bias	Low bias	Probably high bias	Probably high bias	Low bias	Low bias
Vieira Barbosa and Lai [62]	Low bias	Low bias	Probably high bias	Probably high bias	Low bias	Probably high bias
Xu et al. [9]	Low bias	Low bias	Probably high bias	Probably high bias	Low bias	Low bias
Younossi et al. [23]	Low bias	Low bias	Probably high bias	Probably high bias	Low bias	Low bias
Younossi et al. [63]	Low bias	Low bias	Probably high bias	Low bias	Low bias	Low bias
Zeng et al. [26]	Low bias	Low bias	Probably high bias	Low bias	Low bias	Low bias

**Table 2:** ROB analysis of MASLD management methods MASLD: metabolic dysfunction-associated steatotic liver disease; ROB: risk of bias

Study	ROB: sequence generation	ROB: allocation concealment	ROB: blinding of personnel/patient	ROB: blinding of outcome assessor	ROB: incomplete outcome data	ROB: selective outcome reporting
Abid et al. [64]	Low bias	Low bias	Low bias	Low bias	Probably low bias	Low bias
Alam et al. [27]	Low bias	Low bias	Low bias	Low bias	Probably low bias	Probably low bias
Alexander et al. [65]	Low bias	Low bias	Probably high bias	Low bias	Probably low bias	Low bias
Alwahsh and Gebhardt [66]	Low bias	Low bias	Probably high bias	Probably high bias	Low bias	Low bias
Asada et al. [67]	Low bias	Low bias	Low bias	Low bias	Probably low bias	Low bias
Bae et al. [68]	Low bias	Low bias	Low bias	Low bias	Probably low bias	Low bias
Bisaccia et al. [69]	Low bias	Low bias	Probably high bias	Probably high bias	Low bias	Low bias
Bril et al. [70]	Low bias	Low bias	Low bias	Low bias	Probably low bias	Probably low bias
Chalasani et al. [1]	Low bias	Probably high bias	Probably high bias	Probably high bias	Low bias	Low bias
Chen et al. [71]	Low bias	Low bias	Low bias	Low bias	Probably low bias	Low bias
Chongmelaxme et al. [72]	Low bias	Low bias	Probably high bias	Low bias	Low bias	Low bias
Del Bo’ et al. [73]	Low bias	Low bias	Probably high bias	Probably high bias	Low bias	Low bias
Della Pepa et al. [74]	Low bias	Low bias	Probably low bias	Low bias	Probably low bias	Low bias
Ding et al. [75]	Low bias	Probably high bias	Probably high bias	Probably high bias	Low bias	Low bias
Ebadi et al. [76]	Low bias	Low bias	Probably high bias	Probably high bias	Low bias	Low bias
Ferguson and Finck [77]	Low bias	Probably high bias	Probably high bias	Probably high bias	Low bias	Low bias
Fukui et al. [78]	Low bias	Low bias	Low bias	Low bias	Probably low bias	Low bias
Hallsworth et al. [79]	Low bias	Low bias	Low bias	Low bias	Probably low bias	Probably low bias
Hamurcu Varol et al. [80]	Low bias	Low bias	Low bias	Low bias	Probably low bias	Probably low bias
Harrison et al. [81]	Low bias	Low bias	Probably low bias	Low bias	Probably low bias	Probably low bias
Huang et al. [82]	Low bias	Low bias	Probably high bias	Probably high bias	Low bias	Low bias
Kim et al. [83]	Low bias	Low bias	Low bias	Low bias	Probably low bias	Low bias
Koutoukidis et al. [84]	Low bias	Low bias	Probably high bias	Probably high bias	Low bias	Low bias
Kouvari et al. [85]	Low bias	Low bias	Low bias	Low bias	Probably low bias	Low bias
Le et al. [86]	Low bias	Low bias	Low bias	Low bias	Probably low bias	Probably low bias
Lee et al. [28]	Low bias	Low bias	Low bias	Low bias	Probably low bias	Probably low bias
Lian and Fu [87]	Low bias	Low bias	Probably high bias	Probably high bias	Low bias	Low bias
Long et al. [10]	Low bias	Low bias	Probably high bias	Probably high bias	Low bias	Low bias
Loomba et al. [88]	Low bias	Low bias	Probably high bias	Probably high bias	Low bias	Low bias
Maier et al. [89]	Low bias	Probably high bias	Probably high bias	Probably high bias	Low bias	Low bias
Panera et al. [90]	Low bias	Low bias	Low bias	Low bias	Probably low bias	Low bias
Pennisi et al. [91]	Low bias	Low bias	Probably high bias	Probably high bias	Low bias	Low bias
Perumpail et al. [92]	Low bias	Low bias	Probably high bias	Probably high bias	Low bias	Low bias
Raza et al. [93]	Low bias	Low bias	Probably high bias	Probably high bias	Low bias	Low bias
Rinella et al. [94]	Low bias	Probably high bias	Probably high bias	Probably high bias	Low bias	Probably high bias
Romero-Gómez et al. [95]	Low bias	Low bias	Probably high bias	Probably high bias	Low bias	Low bias
Sanyal et al. [96]	Low bias	Low bias	Low bias	Low bias	Low bias	Low bias
Sato et al. [97]	Low bias	Low bias	Probably high bias	Probably high bias	Low bias	Low bias
Semmler et al. [98]	Low bias	Low bias	Low bias	Low bias	Low bias	Low bias
Sinn et al. [99]	Low bias	Low bias	Low bias	Low bias	Low bias	Low bias
Souza et al. [100]	Low bias	Low bias	Probably high bias	Probably high bias	Low bias	Low bias
Stewart et al. [101]	Low bias	Low bias	Low bias	Low bias	Low bias	Low bias
Thoma et al. [102]	Low bias	Low bias	Probably high bias	Probably high bias	Low bias	Low bias
Tincopa and Loomba [103]	Low bias	Low bias	Probably high bias	Probably high bias	Low bias	Low bias
Vilar-Gomez et al. [104]	Low bias	Low bias	Low bias	Low bias	Low bias	Low bias
Vuppalanchi et al. [105]	Low bias	Probably high bias	Probably high bias	Probably high bias	Low bias	Low bias
Wijarnpreecha et al. [106]	Low bias	Low bias	Probably high bias	Probably high bias	Low bias	Low bias
Wong et al. [107]	Low bias	Low bias	Probably low bias	Low bias	Low bias	Probably low bias
Xin et al. [108]	Low bias	Low bias	Low bias	Low bias	Low bias	Probably low bias
Younes and Bugianesi [14]	Low bias	Probably high bias	Probably high bias	Probably high bias	Low bias	Low bias
Younossi et al. [109]	Low bias	Low bias	Probably low bias	Low bias	Probably low bias	Probably low bias
Zelber-Sagi et al. [110]	Low bias	Low bias	Low bias	Low bias	Probably low bias	Low bias

Diagnosis of New MASLD

A total of 43 studies were identified and included in data collection relating to the diagnosis of MASLD, as detailed in Table 3. The multisociety Delphi consensus statement on adopting MASLD on the new nomenclature for NAFLD emphasized the affirmation of the association between NAFLD and cardiometabolic risk factors rather than excluding significant alcohol consumption [3]. Lean MASLD is diagnosed in patients with hepatic steatosis (BMI <25 kg/m^2^ in Asia and <30 kg/m^2^ in the rest of the world) and the presence of at least one cardiometabolic risk factor in the absence of other secondary etiologies of hepatic steatosis [3]. The presence of other drivers of steatosis is termed a combination etiology. Metabolic and alcohol-related/associated liver disease refers to patients meeting criteria for both MASLD and clinically significant alcohol consumption [3,39].

**Table 3:** Comparative analysis of MASLD diagnostic methods from literature 1H-MRS: proton-magnetic resonance spectroscopy; ALD: alcohol-related liver disease; APRI: aspartate-aminotransferase-to-platelet ratio index; BARD: body mass index, aspartate aminotransferase/alanine aminotransferase ratio, and diabetes; BMI: body mass index; CAP: controlled attenuation parameter; CKD: chronic kidney disease; CLD: chronic liver disease; CT: computed tomography; FIB-4: fibrosis-4; FLI: fatty liver index; FLIP: European fatty liver inhibition of progression; FTI: fatty tissue index; HOMA-IR: homeostatic model assessment for insulin resistance; IBD: irritable bowel disease; INR: international normalized ratio; IQR: interquartile ratio; LSM: liver stiffness measurement; LTI: lean tissue index; MAFLD: metabolic dysfunction-associated fatty liver disease; MASLD: metabolic dysfunction-associated steatotic liver disease; MetALD: metabolic and alcohol-related/associated liver disease; MR: magnetic resonance; MRE: magnetic resonance elastography; MRI: magnetic resonance imaging; MRI-PDFF: magnetic resonance imaging -estimated proton density fat fraction; NA: not available; NAFLD: nonalcoholic fatty liver disease; NAS: NAFLD activity score; NASH: nonalcoholic steatohepatitis; NASH CRN: nonalcoholic steatohepatitis clinical research network; NFS: NAFLD fibrosis scores; NITs: noninvasive fibrosis tests; SAF: steatosis activity fibrosis; SLD: steatotic liver disease; SLR: systematic literature review; SROC: summary receiver operator characteristic; T2DM: type 2 diabetes mellitus; TBW: total body water; TE: transient elastography; TG: triglycerides; US: ultrasound; VCTE: vibration-controlled transient elastography; WC: waist circumference; WHR: waist-to-hip ratio; WHtR: waist-to-height ratio

Study	Study design	Study location (country)	Population sociodemographic	Diagnosis method	Outcome investigated	Main findings regarding diagnosis of MASLD
Adams et al. [29]	Prospective study	Australia	242 patients (age, 46.8 ± 12.4 years; male, 60.3%) From three tertiary centers; Sir Charles Gairdner Hospital, Perth, Australia (n = 56); Westmead Hospital, Sydney, Australia (n = 99); and San Giovanni Battista Hospital, Turin, Italy (n = 87)	Biopsy	Simple (APRI, BARD) and complex (Hepascore; FibroTest, BioPredictive, France; and FIB-4) fibrosis models	In NAFLD subjects, noninvasive models have modest accuracy for determining significant fibrosis and have predictive values less than 90% in the majority of subjects. Complex models are more accurate than simple bedside models across a range of fibrosis
Adams et al. [25]	Retrospective study	Germany	65 age-, gender-, and disease-matched underweight patients (age, 30.9 ± 8.9 years) and 65 normal weight controls (age, 30.9 ± 8.9 years)	Abdominal MRI	NAFLD and FIB-4 scores	Patients with extremely low body weight (BMI <17.5 kg/m^2^) showed the highest liver fat content (0.15 units on average; 15%) compared to underweight patients with a BMI of 17.5-18.5 kg/m^2^ (p < 0.05). Furthermore, underweight patients showed slightly increased liver enzymes and liver diameters
Ahadi et al. [2]	Literature review	Iran	Nonobese NAFLD patients	NA	Prevalence, pathophysiology, risk factors, genetic predisposition, diagnosis, treatment, prognosis, and screening of nonobese NAFLD	Liver biopsy is the gold standard method for the diagnosis of NAFLD. However, it is only recommended for patients with an uncertain status, even after employing other modalities. A considerable number of noninvasive tools are helpful in the accurate diagnosis of fibrosis and staging NAFLD, such as NFS and FIB-4
Boursier et al. [30]	Research study	France	938 patients (age, 56.5 ± 12.1 years) with biopsy-proven NAFLD were randomized 2:1 into derivation and validation sets	Biopsy	VCTE, blood fibrosis tests (NAFLD fibrosis score, FIB-4, FibroTest, Hepascore, FibroMeter), and calculation of FibroMeter-VCTE, which combines VCTE results and FibroMeter markers in a single test	For the diagnosis of advanced fibrosis, VCTE was significantly more accurate than the blood tests. The sequential combination of fibrosis tests in the FIB-4-FM^VCTE^ and VCTE-FM^VCTE^ algorithms provides a highly accurate solution for the diagnosis of advanced fibrosis in NAFLD
Castera et al. [31]	Review of literature	France	NAFLD patients	NA	-	The use of noninvasive tests should be tailored according to the setting (primary healthcare, tertiary referral center, and trial) and clinical needs (screening, staging of fibrosis, and follow-up). Regarding detection and grading of steatosis, MRI-PDFF is the most accurate method but appears better suited for assessment and follow-up of selected patients in clinical trials, whereas conventional US, and if no steatosis is shown, CAP, as a point of care technique, could be used as triage in large unselected populations. As for the identification of advanced fibrosis, MRE, TE, FIB-4, and NFS are the most accurate and validated methods
Caussy et al. [32]	Review of literature	United States	NASH patients	NA	MRI-PDFF	Noninvasive, quantitative, precise, and reproducible MRI-PDFF is emerging as a useful biomarker to assess treatment response in the setting of early-phase clinical trials in NASH. It is suitable for quantifying liver fat content. However, it does not assess NASH, fibrosis, inflammation, or other potential endpoints of interest
Cheng et al. [33]	Cross-sectional study	Taiwan	880 subjects from outpatient clinics and the health center of Taipei Tzu Chi Hospital included in the study, lean MAFLD (n = 65; age, 61.1 ± 8.01 years; male, 30.8%) and nonlean MAFLD (n = 329; age, 57.5 ± 10.57 years; male, 57.1%)	US	FTI, LTI, and TBW	In body composition, FTI, LTI, and TBW were lower in lean MAFLD than in nonlean MAFLD patients, but they were comparable with lean healthy controls. Lean MAFLD patients had different metabolic profiles compared with lean healthy controls, but different body composition compared with nonlean MAFLD patients
Cotter and Rinella [34]	Review of literature	United States	NAFLD/NASH patients	NA	Epidemiology, clinical and prognostic features, and diagnostic approach to patients with NAFLD	Liver biopsy is required for NASH, but not for NAFLD, and remains the gold standard for characterizing liver histological alterations in patients with NAFLD. There are two accepted scoring systems for the diagnosis of NASH: the NAS from the NASH CRN and the SAF from the European FLIP consortium. The diagnosis of hepatic steatosis can be made using various imaging modalities: US, CT, VCTE (Fibroscan, Echosens), CAP, and MRI
Denkmayr et al. [35]	Retrospective study	Austria	466 patients with a diagnosis of NAFLD were included in the study. The study cohort consisted of 329 males and 137 females aged between 18 and 75 years	Histology	Lobular inflammation, fasting glucose, and INR	Among lean patients, fasting glucose, INR, and use of thyroid hormone replacement therapy were independent predictors of NASH in a multivariate model. Lean NAFLD patients were characterized by a severe histological picture similar to obese patients but are more progressed compared to overweight patients. Fasting glucose, INR, and the use of thyroid hormone replacement may serve as indicators for NASH in lean patients
Drolz et al. [36]	Retrospective study	Germany	NAFLD patients: n = 368; age, 47; IQR, 35-56 years; females, 57%	Biopsy	Liver fibrosis	This study shows that, among commonly used noninvasive scoring systems, the FIB-4 score is a useful and accurate predictor of advanced fibrosis. FIB-4 scores >1.0 are highly suggestive of advanced fibrosis in NAFLD patients of all BMI categories. NFS tends to overestimate fibrosis in morbidly obese patients
Fu et al. [37]	Prospective study	Hong Kong	709 NAFLD patients at three tertiary referral centers in France, Malaysia, and Hong Kong	Biopsy	Fibrosis	Noninvasive tests of liver fibrosis perform well in nonobese NAFLD patients, and the published cutoffs can be applied in this population. These tests are well suited for initial assessment in the general population or primary care settings
Gibiino et al. [38]	Systematic literature review	Italy	NAFLD patients	NA	Epidemiology, pathogenesis and future directions for the management of NAFLD in patients affected by IBD	Genetic factors, inflammatory signals, and microbiota are key players that could help in understanding the entire pathogenesis of NAFLD, with the aim of defining the multiple expressions of malnutrition
Ha et al. [21]	Systematic literature review	United States	Nonlean NAFLD	NA	Cardiovascular mortality, decompensated cirrhosis, and hepatocellular carcinoma	This study highlights a higher risk of liver-related mortality in patients with lean NAFLD than those with nonlean NAFLD. This finding indicates that further understanding of the pathophysiology, risk factors of adverse outcomes, and genetic and ethnic variabilities of lean NAFLD phenotype is warranted for individualized treatment strategies in lean NAFLD patients. This study includes NAFLD patients diagnosed with biopsy or US
Israelsen et al. [39]	Review of literature	Denmark	NAFLD/ALD/SLD /MetALD patients	NA	Alcohol consumption and metabolic risk factors	For many years, NAFLD and ALD have been considered as two biologically and clinically distinct conditions. According to the newly introduced nomenclature of SLD, patients with moderate excessive alcohol consumption and metabolic risk factors who are diagnosed with SLD will be referred to as having MetALD
Jaruvongvanich et al. [40]	Systematic literature review	United States	NAFLD patients	NA	NFS	High NFS is associated with an increased risk of mortality among patients with NAFLD. This scoring system may be considered an alternative to liver biopsy for the prediction of mortality outcome
Jia et al. [41]	Meta-analysis and SLR	China	NAFLD patients	MRI and TE	MRI-PDFF and TE-CAP	Following a “positive” measurement (over the threshold value) for S1-3, the corresponding post-test probabilities of MRI-PDFF and TE-CAP for the presence of steatosis reached 92% and 88%, respectively, at the pretest probability of 50%. When the values were below the mentioned threshold values (“negative” results), the post-test probabilities of MRI-PDFF and TE-CAP became 5% and 13%, respectively. Both MRI-PDFF and TE-CAP are highly accurate noninvasive methods to grade hepatic steatosis in children and adolescents with NAFLD
Kouvari et al. [42]	Multicenter study	United States	Two Gastroenterology-Hepatology Departments (Greece and Australia) and one Bariatric-Metabolic Surgery Department (Italy). Overall, n = 455 serum samples of patients with biopsy-proven MASLD (n = 374, including 237 patients with MASH) and controls (n = 81) were recruited	Biopsy	NIT	The validation of currently available NITs using biopsy-proven samples provides new evidence for their ability to differentiate between specific disease stages, histological features, and, most importantly, fibrosis grading
Kuchay et al. [43]	Review of literature	India	Lean/nonobese NAFLD	NA	Epidemiology, pathogenesis, and therapeutic management for individuals with either lean or nonobese NAFLD	The majority of prevalence studies for lean NAFLD used US for the detection of liver steatosis, which is a technique that has poor diagnostic accuracy when liver steatosis is below 30%. Therefore, epidemiological studies may have underestimated the true prevalence of NAFLD as an important proportion of individuals with mild liver steatosis may not have been diagnosed as having NAFLD. Indeed, studies using more sensitive methods such as ^1^H-MRS found a higher prevalence rate of NAFLD
Kumar et al. [44]	Retrospective study	India	205 NAFLD patients	Ultrasonography and histology	Noninvasive markers of liver fibrosis and FibroScan	The lean subjects with NAFLD are frequently dyslipidemic. Compared to obese or overweight NAFLD, patients with lean NAFLD have minor or no insulin resistance and appear to have less severe histological disease at presentation. They do not have abdominal obesity, but their BMI was higher than lean healthy control
Lee and Park [45]	Review of literature	South Korea	NAFLD patients	NA	Hepatic steatosis or fibrosis	CT is inaccurate in detecting mild hepatic steatosis and involves a potential radiation hazard, making it inappropriate for assessing hepatic steatosis, especially for longitudinal follow-up of patients with NAFLD. CT, however, may be effective in specific clinical situations, such as the evaluation of hepatic donor candidates for transplantation. MRS is currently the most accurate imaging method used to diagnose hepatic steatosis. MRI, if performed and analyzed correctly, has comparable accuracy to MRS, is more practical, and can cover the entire liver
Li et al. [46]	Research study	China	496 NAFLD cases, obese (BMI ≥25 kg/m^2^) NAFLD group (n = 395) and lean (BMI <25 kg/m^2^) NAFLD group (n = 101)	NA	Alanine aminotransferase, triglycerides, cholesterol, and the blood glucose	Sexual dimorphism exists in lean NAFLD patients, but this trend was most pronounced during the stage of the 40-49-year-old age group and disappeared after entering the 50-59-year-old stage. In lean NAFLD patients, normal levels of TG and blood glucose were more common, and the occurrence of NASH was less common than in obese NAFLD patients
Long et al. [10]	Expert review	United States	NAFLD lean individuals	NA	Liver disease	The initial diagnostic approach should be the same for lean or nonlean individuals with suspected NAFLD. In the case of elevated liver biochemical tests, patients should undergo standard evaluation, including for drug-induced liver injury and chronic liver diseases
Loomba and Adams [47]	Review of literature	United States	NAFLD patients	NA	NITs	Noninvasive assessment of liver fibrosis has become part of routine clinical care for patients with CLD. Serum markers are valuable for screening due to their ease and cost, whereas imaging-based techniques lend themselves as confirmatory tests
Mansour et al. [48]	Research study	United Kingdom	475 consecutive patients with T2DM	NA	Liver stiffness measurement	Embedding a two-tier assessment of liver fibrosis into routine annual diabetes reviews could successfully improve the identification of patients with advanced liver disease by providing a systematic, standardized approach and incorporating it into the management of chronic disease in primary care
Mansour-Ghanaei et al. [49]	Cohort study	Iran	960 individuals who were enrolled in the study: 597 (62.2%) were male, and 363 (37.8%) were female (with an average age of 47.21 ± 7.29 years)	US	BMI, WC, WHR, and WHtR	Anthropometric indices, especially WHtR, as a simple screening tool, seem to be an important criterion for the detection of NAFLD
Martínez-Domínguez et al. [50]	Cross-sectional, case-control study	Spain	300 lean cases with IBD and 80 lean controls without IBD	Liver US, transient elastography, and laboratory tests	Insulin resistance	The overweight/obese and lean IBD groups with MASLD were compared, and the overweight/obese IBD group with MASLD showed higher levels of the homeostatic model assessment of insulin resistance and history of smoking. MASLD prevalence was higher in the lean IBD group compared with the lean non-IBD group, independent of classic metabolic risk factors
Middleton et al. [51]	Multicenter, randomized, double-masked, placebo-controlled, phase 2b trial	United States	113 adults with NASH	Biopsy and MRI	Hepatic steatosis	PDFF estimated by MRI scanners of different field strengths and at different sites accurately classifies grades and changes in hepatic steatosis when histologic analysis of biopsies is used as a reference
Milić et al. [52]	Review of literature	Croatia	NAFLD patients	NA	NA	Obese patients with NAFLD usually do not present with specific symptoms besides a high BMI, metabolic syndrome manifestations, and normal or moderately elevated liver enzyme levels. These patients should be followed in clinical practice for the development of diabetes and HCC via US and alpha-fetoprotein every six months
Navarroza and Wong [53]	Retrospective study	Philippines	663 NAFLD patients	US	Cirrhosis or hepatocellular carcinoma, and AST/ALT	The association of a late diagnosis with older age emphasizes the need for early recognition of risk factors of NAFLD and subsequent screening and intervention in these patients before significant liver injury occurs
Önnerhag et al. [54]	Retrospective study	Sweden	144 NAFLD patients	Biopsy	Noninvasive fibrosis scoring systems (FIB-4-index, NFS, APRI, and BARD score)	Multivariate-adjusted hazard ratios revealed that both the intermediate and high-risk categories of FIB-4 index and NFS could significantly predict metabolic outcomes. All four scoring systems significantly predicted overall mortality in the high-risk category. Noninvasive fibrosis scoring systems, especially NFS and FIB-4-index, can be used to identify patients at risk of future liver-related events, overall mortality, metabolic comorbidities, and CKD
Ooi et al. [55]	Systematic review and meta-analysis	Australia	NAFLD patients	NAFLD fibrosis score	Biomarker panels and elastography techniques	Evidence showed better accuracy of complex biomarker panels (NAFLD fibrosis score: SROC, 0.795-0.813 vs. enhanced liver fibrosis: SROC, 0.962); however, these were poorly validated in obesity. Elastography techniques were better studied and had high diagnostic accuracy (transient elastography: SROC, 0.859; magnetic resonance elastography: SROC, 0.965) but were limited by BMI-dependent failure
Ozturk et al. [56]	Review of literature	United States	Liver fibrosis patients	MR and US elastography	Liver fibrosis	Elastography methods provide LSM as a surrogate quantitative biomarker for fibrosis burden in CLD. Elastography can be performed either with US or MRI
Ratziu et al. [57]	Prospective study	France	51 patients with NAFLD	Liver biopsy	Diagnosis of NASH	Histologic lesions of NASH are unevenly distributed throughout the liver parenchyma. This study demonstrates significant sampling variability of routine liver biopsy in patients with NAFLD. This can result in diagnostic and staging misclassifications in a nonnegligible proportion of patients and is of particular concern as pharmacologic therapies for NASH are emerging
Rinella et al. [3]	Delphi study	United States	The Delphi process was comprised of six components of online data collection (through the Qualtrics platform, Qualtrics International Inc, Provo, UT) and in-person discussions	Histologically or by imaging	Cardiometabolic risk factors	There was consensus to change the definition to include the presence of at least one of five cardiometabolic risk factors. Those with no metabolic parameters and no known cause were deemed to have cryptogenic steatotic liver disease. A new category, outside pure metabolic dysfunction-associated steatotic liver disease, termed MetALD, was selected to describe those with metabolic dysfunction-associated steatotic liver disease who consume greater amounts of alcohol per week (140-350 g/week and 210-420 g/week for females and males, respectively). The new nomenclature and diagnostic criteria are widely supported and nonstigmatizing, and can improve awareness and patient identification
Segura-Azuara et al. [58]	Review of literature	Mexico	MAFLD/NAFLD	Biopsy-free scoring systems	Hepatic steatosis, NASH, and fibrosis diagnosis	The current clinical practice is urged to incorporate biopsy-free scoring systems that demonstrate adequate performance metrics for the accurate detection of patients with MAFLD and underlying conditions or those with contraindications of biopsy
Shida et al. [59]	Research study	Japan	404 patients with NAFLD were divided according to their BMI (nonobese, <25; obese, 25 to <30; and severe obese, ≥30), and were further compared with 253 patients without obesity and NAFLD (non-NAFLD)	Ultrasonography	Liver fat accumulation, hepatic fibrosis, HOMA-IR, and leptin levels	The muscle mass of the nonobese NAFLD group was similar to that of the non-NAFLD group, but muscle steatosis was particularly common among women. Multivariate analysis revealed that the factors contributing to increased liver fat accumulation in the nonobese NAFLD group were visceral fat area, HbA1c, myostatin, and leptin
Sohn et al. [60]	Cross-sectional study	Korea	967 Korean patients with MAFLD. The mean age was 50.8 years, and 869 (90%) patients were male	Ultrasonography	Liver fibrosis	Liver fibrosis in patients with MAFLD varies according to subgroup classification based on BMI and metabolic risk factors. Metabolic dysfunction is an independent risk factor for significant fibrosis in patients with fatty liver, regardless of the etiology of liver disease
Sookoian and Pirola [61]	Systematic review and meta-analysis	Argentina	Lean NAFLD patients	Histology	Fibrosis scores and histological features	Lean-NAFLD patients tend to show less severe histological features compared to overweight/obese-NAFLD patients. Subsequent longitudinal assessment is needed to understand the clinical impact of these findings; however, the significant ~25% increment of mean fibrosis score in overweight/obese patients suggests that obesity could predict a worse long-term prognosis
Vieira Barbosa and Lai [62]	Letter to the editor	United States	NAFLD patients	NA	Liver fibrosis assessment	NAFLD in patients with type 2 diabetes using noninvasive scoring systems such as the FIB-4 score and VCTE. The proposed algorithm involves a first-step annual FIB-4 score followed by VCTE for those with indeterminate or high-risk scores (FIB-4 >1.3). Low-risk patients (FIB-4 <1.3 or VCTE <8 kPa) can be followed up by primary care providers for lifestyle changes and yearly calculation of FIB-4, while high-risk patients (FIB-4 >1.3 and VCTE >8 kPa) should be referred to liver specialized clinics for further assessment and evaluation
Xu et al. [9]	Review of literature	China	Lean NAFLD patients	NA	Environmental and genetic susceptibility, and epigenetic regulation	The approach for lean NAFLD diagnosis is identical to any patient with NAFLD. Excluding excessive alcohol intake and other causes of hepatic steatosis and damage, the amount of intrahepatic fat content was assessed through US, CT, or MR. But these methods used in chronic liver disease have limitations for sensitivity and accuracy, including lean NAFLD
Younossi et al. [23]	Review of literature	Italy	NAFLD patients	Biopsy	Disease progression prediction, risk factors, and prevention	The large number of patients with NAFLD with the potential for progressive liver disease creates challenges for screening, as the diagnosis of NASH necessitates invasive liver biopsy. Furthermore, individuals with NAFLD have a high frequency of metabolic comorbidities and could place a growing strain on healthcare systems from their need for management
Younossi et al. [63]	Descriptive study	United States	NAFLD patients	US	Prevalence of NAFLD	Multivariate analysis showed that lean NAFLD was independently associated with younger age, female sex, and a decreased likelihood of having IR and hypercholesterolemia (p values of 0.05). Additionally, multivariate analysis showed that NASH was independently associated with being Hispanic, having a younger age, and having components of metabolic syndrome such as hypertension (p values of 0.05). Lean individuals with NAFLD have a different clinical profile than overweight, obese individuals with NAFLD
Zeng et al. [26]	Retrospective study	China	2,715 participants were included in the study	Ultrasonography	FLI	NAFLD is not uncommon in lean Chinese adults, even with a normal WC. Metabolic factors, rather than genetic factors, may play important roles in the development of NAFLD in this population. A lower cutoff value of the FLI score in screening for NAFLD should be used for lean Chinese adults with a normal WC

Lean MASLD is often asymptomatic but can present with fatigue, malaise, or mild abdominal discomfort [23,52,53]. Due to its mostly asymptomatic nature, it is mostly diagnosed incidentally with abdominal imaging [2]. Patients with lean MASLD have a lower waist-to-hip ratio, waist circumference, and visceral fat compared to patients with obese MAFLD [59]. Again, compared to obese MASLD patients, lean MASLD patients are often younger and have lower levels of fasting glucose, hemoglobin A1c (HbA1c), blood pressure, and homeostasis model assessment insulin resistance index (HOMA-IR) [26,44,50,53,63]. In contrast, studies have shown that patients with lean MASLD have higher waist circumference, mean BMI, body fat, fasting glucose, deranged lipid panel, blood pressure, and HbA1c relative to lean non-MASLD groups [33,44,49,59].

Mild elevations in alanine aminotransferase (ALT) and aspartate aminotransferase (AST) with AST/ALT ratio <1 can be seen, but levels might be normal in some cases, suggesting their poor reliability as markers of hepatic steatosis [9,52]. Some studies have identified higher median ALT levels in nonlean MASLD patients than those with lean MASLD [33,46,53,63]. However, in a lean MASLD cohort with irritable bowel disease, Adams et al. found higher average values of liver enzymes in the underweight group than in the normal-weight group [25].

Ultrasonography, computed tomography (CT), and magnetic resonance imaging (MRI) can detect hepatic steatosis. No significant differences exist in diagnosing MASLD on imaging in lean and obese subjects [2,52]. Ultrasound (US) has a sensitivity ranging from 80% to 100% in detecting hepatic steatosis. However, this declines in the presence of coexisting chronic liver disease and hepatic fibrosis [45]. The limitation of CT scan in evaluating MASLD lies in its low accuracy in detecting mild hepatic steatosis, making it unsuitable for screening as a significant proportion of patients have mild fatty infiltration [45]. MRI is superior to CT and US in evaluating hepatic steatosis. There is emerging evidence that MRI and magnetic resonance (MR) spectroscopy-derived proton density fat fraction (PDFF) may be a more accurate and reproducible measure of quantifying hepatic fat content than the traditional gold standard of histology, which is fraught with interobserver variability and invasive complications [32,41,45]. Middleton et al., in a multicenter RCT in patients with NASH, found MRI-PDFF to assess hepatic steatosis grade accurately and changes with histology as a reference after treatment with obeticholic acid (OCA) versus placebo [51]. Satkunasingham et al., in a retrospective study involving living liver donor candidates, found MR-PDFF as accurate at excluding clinically relevant fatty liver, precluding the utility for liver biopsy [111].

Detection of hepatic steatosis alone is inadequate for holistic patient management in MASLD. Early detection of liver fibrosis, which is the primary determinant of progression to cirrhosis, HCC, cardiovascular morbidity, and mortality, is essential [2,43]. A meta-analysis by Sookoian and Pirola found an increment of 25% in mean fibrosis and severe histological features in overweight/obese subjects compared to lean/nonobese MASLD counterparts [61].

Noninvasive fibrosis scoring systems; including fibrosis-4 (FIB-4); NAFLD fibrosis score (NFS); BMI, AST/ALT ratio (BARD) score; and AST-to-platelet ratio index; have all been validated in MASLD [42,58]. Elevated NFS and FIB-4 scores have been associated with increased risks of adverse liver events, metabolic complications, and mortality [40,54], suggesting obesity is a poor long-term outcome in MASLD [61]. Noninvasive serological tests such as enhanced liver fibrosis score (ELF) and Hepascore are more accurate than the abovementioned simple models at predicting advanced fibrosis as they include direct biomarkers of fibrosis [29]. These serological tests have not been validated in lean/nonobese MASLD patients [43]. However, their performance has been comparable and even higher at detecting advanced fibrosis in lean versus obese MASLD in some studies [36,37].

Transient elastography can assess liver fibrosis and fat content without being confounded by steatosis [38]. Elastography is more accurate than noninvasive fibrosis tests and scoring systems [47] but may be limited as one becomes increasingly obese [47,55]. MR elastography is superior to US elastography. It is not significantly limited by obesity and has less intra- and interobserver variabilities [56]. However, its utility is limited by cost and availability [47,56]. The combination of fibrosis scoring systems and elastography, concurrently or sequentially, has shown promise as highly accurate tools in assessing MASLD, fibrosis, and mortality risk [30,31,47,62]. Boursier et al., in a randomized trial, found the sequential combination of FIB-4 followed by FibroMeter (Echosens, France) vibration-controlled transient elastography (VCTE) to be highly accurate (90%) at diagnosing advanced fibrosis with biopsy being required in only 20% for diagnosis conformation [30]. A study utilizing a sequential combination with FIB-4 followed by transient elastography at two primary care centers in England enhanced the diagnosis of advanced fibrosis sevenfold, with an odds ratio (OR) of 6.71 (2.0-22.7) and a p value of 0.0022, compared to standard care [48]. Additional studies are required to validate such combination approaches in lean and general MASLD populations.

While invasive, liver biopsy remains the gold standard for confirming MASLD and assessing the grade (degree of inflammation) and stage (fibrosis) [34]. Disadvantages of liver biopsy include expertise, sampling errors, intra- and interobserver variabilities, morbidity associated with its invasiveness, and cost implications [34,57] and are indicated only in the presence of uncertainty from standard tests regarding alternative/contributory etiologies and liver fibrosis staging in patients with lean MASLD [10]. Histological findings from patients with lean MASLD have shown similar severe features compared with obese MASLD patients [21,35,60].

Management of Lean MASLD

General management: A total of 52 studies relating to managing lean MASLD were identified, as shown in Table 4. Lifestyle modifications, including diet and increased physical activity aimed at weight loss, remain the foundation of management [10,94,95]. Some authors have used the term metabolically obese normal weight to describe patients with lean MASLD in terms of their increased adipocyte mass, insulin resistance, and hypertriglyceridemia, which can be remedied through a hypocaloric diet and/or weight loss [1,75]. Increased physical activity and exercise, irrespective of BMI and weight loss, have been associated with a reduced risk of MASLD development and improvement in the biochemical, histological, and cardiometabolic profiles of patients with MASLD [67,68,79]. The 2023 American Gastroenterological Association (AGA) guidelines on lean MASLD recommend a target of 3%-5% weight loss through increased physical activity and dietary interventions [10]. Sustaining the gains of weight loss remains elusive to many patients with MASLD, highlighting the role of a multidisciplinary approach, including nutrition services and psychological, family, and social support systems [94,101,102]. An RCT from China utilizing 12 months of lifestyle interventions resulted in the resolution of MASLD in half of the nonobese patients through 3%-5% weight loss compared with the same outcome in the obese group with 7%-10% weight loss. The weight loss gains were more likely to be maintained by the sixth year by the nonobese group [107]. Alam et al., in a prospective study from Bangladesh, showed that weight reduction of any magnitude resulted in significant improvement in steatosis, nonalcoholic fatty liver disease activity score (NAS) scores, and histological profile in patients with lean and nonlean NASH after one year [27]. A Turkish study utilizing intensive lifestyle modifications, including diet and increased physical activity with an ensuing 5% weight loss, resulted in the resolution of hepatic steatosis in 57% of patients with lean MASLD after eight weeks [80]. Other studies have shown that weight loss through physical activity and other nutritional interventions has a dose-dependent association with a resolution of hepatic steatosis in patients with lean and nonlean MASLD [84,99].

**Table 4:** Comparative analysis of MASLD management methods from literature 1H-MRS: proton magnetic resonance spectroscopy; ADIPO-IR: adipose tissue insulin resistance; ALT: alanine aminotransferase; AOR: adjusted odds ratio; APRI: aspartate aminotransferase-to-platelet index; ASCVD: atherosclerotic cardiovascular disease; AST: aspartate aminotransferase; BMI: body mass index; CI: confidence interval; CrI: credible interval; CRP: C-reactive protein; CT: computed tomography; CV: cardiovascular; CVD: cardiovascular disease; DM: diabetes mellitus; FIB-4: fibrosis-4; FLI: fatty liver index; GGT: gamma-glutamyl transferase; GWAS: genomewide association studies; HCC: hepatocellular carcinoma; HOMA-IR: homeostatic model assessment for insulin resistance; HR: hazard ratio; HRI: hepatorenal index; HSI: hepatic steatosis index; HXT: hydroxytyrosol; ICERs: incremental cost-effectiveness ratios; IHTG: intrahepatic triglyceride; ION: index of NASH; LFE: liver fat equation; LFS: liver fat score; MACCE: major adverse cardiovascular and cerebrovascular events; MASLD: metabolic dysfunction-associated steatotic liver disease; MD: Mediterranean diet; MONW: metabolically obese, normal-weight; MRI-PDFF; magnetic resonance imaging proton density fat fraction; MUFAs: monounsaturated fatty acids; NA: not available; NAFLD: nonalcoholic fatty liver disease; NAS: nonalcoholic fatty liver disease activity score; NASH: nonalcoholic steatohepatitis; OR: odds ratio; PA: physical activity; PUFAs: polyunsaturated fatty acids; QALYs: quality-adjusted life years; RR: risk ratio; T2DM: type 2 diabetes mellitus; TC: total cholesterol; US: ultrasound; VAI: visceral adiposity index; Vs: velocity of shear wave; WC: waist circumference; γ-GTP: γ-glutamyl transpeptidase

Study	Study design	Study location (country)	Population sociodemographic	Diagnosis method	Outcome investigated	Main findings regarding the management of MASLD
Abid et al. [64]	Cross-sectional study	Israel	31 patients (age: 43 ± 12 years; 50% males) with NAFLD and risk factors for metabolic syndrome and 29 patients (age: 41 ± 11 years; 47% males) with NAFLD and without risk factors for metabolic syndrome, and 30 gender- and age-matched (age: 40 ± 10 years; 49% males) individuals without NAFLD. Patients were recruited from the liver unit at the Ziv Medical Center in Safed, Israel	US examination	Insulin resistance, inflammation, oxidant-antioxidant markers, soft drink consumption, and the presence of NAFLD	The study found that 80% of patients with NAFLD had excessive intake of soft drink beverages (>500 cm^3^/day) compared to 17% of healthy controls. The NAFLD group consumed five times more carbohydrates from soft drinks than healthy controls (40% vs. 8%, p < 0.001). The most common soft drinks were Coca-Cola (regular: 32%; diet: 21%) followed by fruit juices (47%). Logistic regression analysis showed that soft drink consumption is a strong predictor of fatty liver (odds ratio: 2.0; p < 0.04) independent of metabolic syndrome and CRP level. This paper just mentioned the role of sugar-sweetened beverages in NAFLD but did not mention its management
Alam et al. [27]	Prospective study	Bangladesh	15 lean (age, 34.80 ± 8.66 years; M/F, 6/9) and 16 nonlean (age, 37.88 ± 5.83, M/F, 6/10) histologically proven NASH patients	Liver biopsies with histology	Weight reduction, steatosis, ballooning, lobular inflammation, NAS, and fibrosis	A weight reduction strategy of one year significantly improved the histologic activity of the liver in both lean and nonlean NASH patients. In the lean/nonlean group, any amount of weight reduction, ≥5% weight reduction, and ≥7% weight reduction was found in, respectively, 8/11, 5/6, and 2/6 patients. In both lean and nonlean groups, weight reduction of any amount was associated with a significant reduction of steatosis, ballooning, and NAS, except lobular inflammation and fibrosis. In both groups, weight reduction of ≥5% was associated with a significant reduction in NAS only. However, significant improvement in NAS was noted with ≥7% weight reduction in nonlean group only
Alexander et al. [65]	Matched cohort study	United Kingdom	NAFLD/NASH patients aged ≥18 at diagnosis and had medical records available for ≥12 months	Coded NAFLD/NASH diagnosis from European primary care databases representing the UK, the Netherlands, Italy, and Spain	Cirrhosis or hepatocellular carcinoma	Out of 18,782,281 adults, we identified 136,703 patients with coded NAFLD/NASH. Coded NAFLD/NASH patients were more likely to have diabetes, hypertension, and obesity than matched controls. HR for cirrhosis in patients compared to controls was 4.73 (95% CI, 2.43-9.19), and for HCC, 3.51 (95% CI, 1.72-7.16). HR for either outcome was higher in patients with NASH and those with high-risk FIB-4 scores. The strongest independent predictor of a diagnosis of HCC or cirrhosis was a baseline diagnosis of diabetes. Further, better biomarkers are needed to identify those at risk more precisely. This study did not clearly mention the management of NAFLD
Alwahsh and Gebhardt [66]	Review of literature	United Kingdom	NAFLD patients	NA	Obesity, dyslipidemia, and insulin resistance	Still, lifestyle modification, lessening the consumption of fructose-containing products, and promoting physical exercise are major measures against NAFLD. Finally, promising drugs against fructose-induced insulin resistance and hepatic dysfunction that are emerging from studies in rodents are reviewed but need further validation in human patients
Asada et al. [67]	Cross-sectional study	Japan	45 patients with NAFLD (20 patients without PA; age, 61.8 ± 2.5 years and 25 patients with increased PA; age, 56.5 ± 2.6 years)	Diagnostic imaging and diagnostic criteria issued by the Japanese Society of Gastroenterology	Hepatic inflammation, and blood and biochemical parameters	Results suggested that hepatic inflammation improvement was accompanied by increased PA but not decreased body weight. In the PA increase group, aspartate aminotransferase, alanine aminotransferase, and γ-guanosine triphosphate levels were all significantly lower at follow-up than they were at baseline. Body weight did not change significantly from baseline to follow-up in both groups
Bae et al. [68]	Cross-sectional study	Republic of Korea	Nonexercise (n = 59,392; age, 41.3 ± 68.3 years; males, 54.6%) and exercise (n = 12,967; age, 45.2 ± 68.9 years; males, 50.2%) groups; Koreans population	Ultrasonography	Liver enzymes	The risk for NAFLD was significantly reduced in the exercise group with age- and sex-adjusted ORs of 0.53-0.72 for all BMI deciles except at BMI categories of 19.6 and 20.7-21.6 kg/m^2^. While no difference was seen in BMI between subjects in the exercise and nonexercise groups across the BMI deciles, the values of body fat percentage and metabolic risk factors differed. Among NAFLD patients, subjects in the exercise group had a lower risk of having elevated liver enzymes with multivariable AOR of 0.85 (95% CI, 0.74-0.99, for AST) and 0.74 (95% CI, 0.67-0.81, for ALT) than did subjects in the nonexercise group
Bisaccia et al. [69]	Systematic review and meta-analysis	Italy	A total study population of 10,592,851 individuals (mean age, 53 ± 8; male sex, 50%; NAFLD2, 9%)	NA	Cardiovascular morbidity and mortality	Compared with nonlean NAFLD, lean NAFLD was associated with increased CV mortality (OR: 1.50; 95% CI, 1.1-2.0), but similar all-cause mortality and risk of MACCE. Patients with NAFLD are at high risk of CV consequences independent of their body mass status, and lean phenotypes should not be overlooked in risk assessment for CV disease prevention
Bril et al. [70]	Prospective study	United States	Biopsy-proven NASH (52 with type 2 diabetes and 49 with prediabetes)	Biopsy	Reduction in the nonalcoholic fatty liver disease activity score of 2 points or more, NASH resolution, individual histologic components, intrahepatic triglyceride content, and insulin sensitivity	Pioglitazone is effective in patients with and without type 2 diabetes. Resolution of NASH was achieved in 44% of patients with type 2 diabetes vs. 26% without diabetes
Chalasani et al. [1]	Practice guidance	United States	NA	NA	NA	This paper mentioned the management of NAFLD through lifestyle modifications, including weight loss, physical activity, and dietary changes, Pharmacotherapy including vitamin E and pioglitazone, bariatric surgery, monitoring for disease progression, and multidisciplinary care, including collaboration between hepatologists, endocrinologists, dietitians, and other healthcare professionals
Chen et al. [33]	Cross-sectional study	China	A cohort of 233 patients with T2DM from Xiamen, China	Ultrasonography	FIB-4 score	Screening and management of NAFLD, especially for those with underweight or normal weight, should be strengthened from the perspective of improving the prevention and management of cancer in patients with T2DM
Chongmelaxme et al. [72]	Cost-utility analysis study	Thailand	Thai patients with NAFLD	NA	Number of cirrhosis and HCC cases, life expectancy, QALYs, lifetime costs, and the ICERs	When compared with usual care, a weight reduction program can prevent cirrhosis and HCC cases by 13.91% (95% CrI, 0.97-20.59) and 2.12% (95% CrI, 0.43-4.56), respectively; pioglitazone can prevent cirrhosis and HCC cases by 9.30% (95% CrI, -2.52 to 15.24) and 1.42% (95% CrI, -0.18 to 3.74), respectively; and vitamin E can prevent cirrhosis and HCC cases by 7.32% (95% CrI, -4.64 to 15.56) and 1.12% (95% CrI, -0.81 to 3.44), respectively
Del Bo’ et al. [73]	Systematic review and meta-analysis	Italy	NAFLD patients: adult participants (age ≥18 years) and overweight and obese adults (BMI ≥25 kg/m^2^)	NA	GGT, ALT, TC, WC, and liver fibrosis	MD might reduce indirect and direct outcomes linked with NAFLD severity, such as TC, liver fibrosis, and WC, although it is important to consider the variations across trials
Della Pepa et al. [74]	Multicenter randomized controlled trial	Italy	Patients with T2DM (n = 195) aged 50-75 years	NA	Indirect indices of NAFLD (LFE, HSI, and ION) and insulin resistance (HOMA-IR, VAI, and ADIPO-IR)	Indices of NAFLD improved after pioglitazone, but not after sulfonylureas. One-year treatment with pioglitazone, even at low dosage, significantly improved liver steatosis and inflammation, systemic and adipose tissue insulin resistance in patients with T2DM
Ding et al. [75]	Review	Singapore	NA	NA	NA	MONW is a unique phenotype that may or may not respond to current therapies that have been effective in obese persons
Ebadi et al. [76]	Systematic review and meta-analysis	Canada	NAFLD patients	Serum biomarkers of liver injury or imaging studies (i.e., US and computed tomography), elastography, or liver histological evaluation	Steatosis and fibrosis severity classification	There was no association between coffee consumption and NAFLD incidence (RR, 0.88; 95% CI, 0.63-1.25; p = 0.48) or NAFLD prevalence (RR, 0.88; 95% CI, 0.76-1.02; p = 0.09). The meta-analysis confirms the protective role of regular coffee consumption on the risk of significant liver fibrosis
Ferguson and Finck [77]	Literature review	United States	NAFLD and T2DM patients	NA	NASH biomarkers	Due to the close association between NAFLD and T2DM, many agents that are currently prescribed for hyperglycemia have yielded positive results on NASH biomarkers. Experimental agents that target aspects of intermediary metabolism have also proven beneficial to varying degrees, but adverse effects might limit their use
Fukui et al. [78]	Retrospective study	Japan	38 NAFLD patients (age, 62.0 ± 11.6 years; male/female, 10/28)	Liver biopsy	Liver enzyme levels (AST, ALT, and γ-GTP), noninvasive scoring systems of hepatic FIB-4 index and APRI, and liver stiffness (Vs) measured by acoustic radiation force impulse elastography	One year of vitamin E treatment improved noninvasive fibrosis scores and liver stiffness in NAFLD patients
Hallsworth et al. [79]	Cohort study	United Kingdom	21 people with NAFLD	NA	NAFLD fibrosis score	Resistance exercise specifically improves NAFLD independent of any change in body weight
Varol et al. [80]	Research study	Turkey	35 consecutive patients (14 lean and 21 obese) with NAFLD (>18 years of age)	Hepatic steatosis detected by transient elastography	Hepatic steatosis	5% body weight loss is effective in both obese and lean patients, resulting in a similar NAFLD remission
Harrison et al. [81]	Randomized, double-blind, placebo-controlled study	United States	185 patients (41 in the placebo and 84 in the resmetirom group). In the placebo, the age was 47·3 ± 11·7 years, and the percentage of males was 59%, while in resmetirom, the age was 51.8 ± 10.4 years, and the percentage of males was 45%	Biopsy and MRI-PDFF	Hepatic fat	Resmetirom treatment resulted in a significant reduction in hepatic fat after 12 weeks and 36 weeks of treatment in patients with NASH
Huang et al. [82]	Systematic review and meta-analysis	China	NAFLD patients and nonlean NAFLD patients	US, biopsy	All-cause death	Lean patients with NAFLD had a higher risk of all-cause death than nonlean patients. BMI should not be used as a criterion to determine whether further observation and therapy of patients with NAFLD are warranted. Among the lean NAFLD population, all-cause mortality was 13.3 (95% CI, 6.7-26.1) per 1,000 person-years, 3.6 (95% CI, 1.0-11.7) for liver-related mortality, and 7.7 (95% CI, 6.4-9.2) for cardiovascular-related mortality
Kim et al. [83]	Retrospective study	Korea	4,786 subjects (1,740 men and 3,046 women) subjects aged ≥40 years	NAFLD score (CNS) and the NAFLD LFS	Cardiovascular risk	Subjects with lean NAFLD had a significantly higher ASCVD score and prevalence of high risk for ASCVD than those with obese NAFLD. Similarly, lean subjects with significant liver fibrosis had a higher probability of ASCVD than obese subjects in the subpopulation with NAFLD
Koutoukidis et al. [84]	Systematic review and meta-analysis	United Kingdom	People with NAFLD	NA	Changes in blood, radiological, or histological biomarkers of liver disease	There was evidence of a dose-response relationship with liver inflammation, ballooning, and resolution of NAFLD or NASH, but limited evidence of a dose-response relationship with fibrosis or NAFLD activity score. Clinically significant improvements in NAFLD are achieved even with modest weight loss, but greater weight loss is associated with greater improvements
Kouvari et al. [85]	Prospective cohort study	Athens, Greece	1,514 men and 1,528 women (>18 years old) free-of-CVD at baseline	-	MedDiet score and fatal/nonfatal CVD incidence	MedDiet score was inversely associated with steatosis and fibrosis, e.g., in the case of the TyG index, the OR of the third vs. first MedDiet score tertile was 0.53 (95% CI, 0.29-0.95), and the associations persisted in multiadjusted models. MedDiet protected against diabetes and CVD prospectively among subjects with NAFLD
Le et al. [86]	Cross-sectional analysis	United States	40,323 United States adults; 5,690 had T2DM. Of 4,438 patients without other causes of fatty liver, 3,140 (71%) had concurrent suspected NAFLD. Mean age was 59.4 years and 47.9% were male	NA	NA	Pioglitazone use peaked at 20% in 2005-2006, then declined to 4.1% in 2013-2014. Patients with suspected NAFLD were not more likely to receive pioglitazone; 3.8% of patients received the drug in 2015-2016
Lee et al. [28]	Large population-based study	South Korea	11,593,409 subjects from the National Health Information Database of the Republic of Korea entered in 2010 and followed up until 2016	FLI and significant liver fibrosis	Liver fibrosis	The use of statin was associated with a reduced risk of NAFLD development (AOR, 0.66; 95% CI, 0.65-0.67) and was independent of associated DM (with DM: AOR, 0.44; 95% CI, 0.41-0.46; without DM: AOR, 0.71; 95% CI, 0.69-0.72). The use of statins reduced the risk of significant liver fibrosis (AOR, 0.43; 95% CI, 0.42-0.44), independent of DM (with DM: AOR, 0.31; 95% CI, 0.31-0.32, without DM: AOR, 0.52; 95% CI, 0.51-0.52)
Lian and Fu [87]	Meta-analysis	China	NAFLD patients with prediabetes or type 2 diabetes mellitus	NA	Steatosis grade, inflammation grade, ballooning grade, and fibrosis stage	Compared with placebo, pioglitazone significantly improved steatosis grade, inflammation grade, and ballooning grade, while in the fibrosis stage, there was no significant improvement in pioglitazone compared with placebo. In addition, pioglitazone can also improve blood glucose and liver function
Long et al. [10]	Expert review	United States	NAFLD patients	Serum indices (NAFLD fibrosis score and FIB-4) and imaging techniques (transient elastography and MRI elastography)	Disease progression, complications, and mortality	Lifestyle intervention, including exercise, diet modification, and avoidance of fructose and sugar-sweetened drinks, to target a modest weight loss of 3%-5% is suggested for lean patients with NAFLD. Screening for NAFLD should be considered for individuals over the age of 40 with T2DM
Loomba et al. [88]	Expert review	United States	NAFLD patients	NA	NA	Obesity remains an important risk factor for NAFLD and NAFLD-associated HCC, and weight loss interventions are strongly recommended to improve NAFLD-related outcomes
Maier et al. [89]	Literature review	United States	Lean NAFLD	NA	NA	Patients with lean NAFLD frequently have less severe metabolic abnormalities than obese NAFLD
Panera et al. [90]	Research study	Italy	TGF-β-activated LX-2 cells as an in vitro model, and carbon tetrachloride plus a Western diet as a mice model	Liver biopsy	Expression of profibrogenic genes and reduction of fibrosis	Supplementation with HXT and vitamin E can be a potential therapeutic approach to improve NAFLD-related liver fibrosis, thus reducing the risk of disease progression
Pennisi et al. [91]	Systematic reviews and meta-analyses	Italy	Nonalcoholic steatohepatitis patients	NA	NASH resolution	Semaglutide (p score = 0.906), pioglitazone alone (score 0.890), and plus vitamin E (0.826) had the highest probability of being ranked the most effective intervention for NASH resolution without worsening of fibrosis, while aldafermin (0.776), lanifibranor (0.773), and obeticholic acid (0.771) had the highest probability to achieve ≥1 stage of fibrosis improvement without worsening of NASH
Perumpail et al. [92]	Systematic review of literature	United States	NAFLD patients	NA	NAFLD-related outcomes	Most studies concluded and recommended a reduction in the intake of saturated and trans fatty acids, carbohydrates, and animal-based protein, and an increased intake of PUFAs, MUFAs, plant-based proteins, antioxidants, and other nutrients was recommended. The MedDiet and paleo diet both seem to be promising schemes for NAFLD patients to follow. Exercise was also encouraged, but the type of exercise did not affect its efficacy as an NAFLD treatment when the duration was consistent
Raza et al. [93]	Review of literature	India	NAFLD/NASH patients	NA	NA	While there is no FDA-approved medication for NAFLD/NASH, dietary and lifestyle intervention is the mainstay of treatment. Several medications are in the pipeline of therapy for NASH, holding promise for successful therapy in the future. As of now, first-line drugs such as pioglitazone and vitamin E remain the strategy for disease management in patients
Rinella et al. [94]	Practice guide	United States	NAFLD patients	NA	NA	Lifestyle modifications, such as weight loss and increased physical activity, are recommended as first-line therapy for patients with NAFLD. Multidisciplinary care involving primary care physicians, endocrinologists, and hepatologists is recommended for the management of NAFLD
Romero-Gómez et al. [95]	Review of literature	Spain	NAFLD patients	NA	NASH resolution or fibrosis improvement	Lifestyle intervention can be useful across all the spectra of NAFLD patients. Losing weight decreases cardiovascular/diabetes risk and also regresses liver disease. Weight reductions of 10% are required to induce near-universal NASH resolution or fibrosis improvement by at least one stage. However, modest weight losses (>5%) also produce important benefits for NAS and its components. In addition, to improve the success of this intervention, we need to explore, beyond total calories and type of weight loss diet, the role of micro- and macronutrients, evidence-based benefits of physical activity, and exercise, finally supporting these modifications through established behavior change models and techniques. The Mediterranean diet can reduce liver fat even without weight loss and is the most recommended dietary pattern in NAFLD
Sanyal et al. [96]	Research article	United States	247 adults (age, 46.3 ± 11.9 years, female, 60%) with nonalcoholic steatohepatitis and without diabetes	Standardized grading system for steatosis (on a scale of 0-3), lobular inflammation (on a scale of 0-3), and hepatocellular ballooning (on a scale of 0-2)	Improvement in histologic features of nonalcoholic steatohepatitis	Vitamin E therapy, compared with placebo, was associated with a significantly higher rate of improvement in nonalcoholic steatohepatitis (43% vs. 19%, p = 0.001), but the difference in the rate of improvement with pioglitazone as compared with placebo was not significant (34% and 19%, respectively; p = 0.04)
Sato et al. [97]	Meta-analysis	Japan	NAFLD/NASH patients	Histology or ultrasonography	Resolution of steatosis, inflammation, and hepatocellular ballooning	Vitamin E significantly reduced AST of -19.43 U/L, ALT of -28.91, ALP of -10.39 U/L, steatosis of -0.54, inflammation of -0.20, and hepatocellular ballooning of -0.34 compared with the control group in NAFLD patients. Vitamin E treatment with NASH adult patients showed obvious reductions in not only AST of -13.91 U/L, ALT of -22.44 U/L, steatosis of -0.67, inflammation of -0.20, but also fibrosis of -0.30 compared with control treatment
Semmler et al. [98]	Single-center cross-sectional study	Austria	3,043 subjects (cohort I) and 1,048 subjects (cohort II)	Colonoscopy	NA	NAFLD is frequently observed in lean individuals, with a prevalence of 20%-40%. NAFLD in lean individuals shows a strong association with metabolic syndrome and its components, especially glycemic dysregulation. NAFLD is associated with an increased cardiovascular risk in lean subjects
Sinn et al. [99]	Longitudinal study	South Korea	16,738 adults (average age, 50.5 years; lean NAFLD, 2,383 participants) with NAFLD	Ultrasonography	Resolution of fatty liver	There was a dose-dependent association between weight reduction and fatty liver resolution in both lean and overweight/obese NAFLD patients
Souza et al. [100]	Systematic review and meta-analysis	Brazil	Lean (BMI <25 kg/m^2^ or <23 kg/m^2^ in Asians) and nonlean (BMI ≥25 kg/m^2^ or ≥23 kg/m^2^ in Asians) NAFLD individuals	NA	Cancers	Lean NAFLD patients have an increased risk of liver, pancreatic, and colorectal cancers compared to nonlean NAFLD patients, emphasizing the need to explore tailored cancer prevention strategies for this specific patient group
Stewart et al. [101]	Research study	United States	Fifty-eight overweight/obese (BMI ≥25) adults with biopsy-proven NAFLD	Biopsy	Neuroticism and conscientiousness	Six-month change in weight was nonsignificant and was not associated with baseline readiness for change. Depression, low conscientiousness, and high neuroticism were associated with higher weight at six-month follow-up with small-to-large effect sizes. Integrated multidisciplinary approaches that address psychiatric needs and provide behavioral support for weight loss may help patients with NAFLD implement sustained lifestyle changes
Thoma et al. [102]	Systematic review	United Kingdom	NAFLD patients	Histological examination of biopsies, 1H-MRS, CT; US, and/or blood concentrations of ALT and/or AST	Steatosis, histological evidence of inflammation and fibrosis, and glucose control/insulin sensitivity	Studies consistently showed reductions in liver fat and/or liver aminotransferase concentration, with the strongest correlation being with weight reduction. Of the five studies reporting changes in histopathology, all showed a trend toward reduction in inflammation; in two studies, this was statistically significant. Changes in fibrosis were less consistent, with only one study showing a significant reduction. The majority of studies also reported improvements in glucose control/insulin sensitivity following intervention. Lifestyle modifications leading to weight reduction and/or increased physical activity consistently reduced liver fat and improved glucose control/insulin sensitivity
Tincopa and Loomba [103]	Review of literature	United States	NASH and NAFLD patients	Liver biopsy, NAS ≥4, and fibrosis stage ≥2	NA	With emerging pharmacotherapy, noninvasive tests are required to track treatment response. There is an unmet need for noninvasive tests to assess risk for clinical outcomes, including progression to cirrhosis, hepatic decompensation, liver-related mortality, and overall mortality. This review examines the advances in noninvasive tests to diagnose and monitor NAFLD and NASH
Vilar‐Gomez et al. [104]	Retrospective study	United States	236 patients	Biopsy	All-cause mortality (including liver- and non-liver-related) or liver transplantation	Vitamin E use was associated with improved clinical outcomes in patients with NASH and bridging fibrosis or cirrhosis
Vuppalanchi et al. [105]	Review of literature	United States	NASH patients	NA	NA	Glycemic control, lipid profile, and weight loss have beneficial effects on NASH
Wijarnpreecha et al. [106]	Systematic review and meta-analysis	United States	NAFLD patients	NA	Liver fibrosis and NAFLD	A significantly decreased risk of NAFLD among coffee drinkers and a significantly decreased risk of liver fibrosis among patients with NAFLD who drank coffee on a regular basis
Wong et al. [107]	Single-blind randomized controlled trial	Hong Kong	154 community NAFLD	1H-MRS and transient elastography	Remission of NAFLD at month 12. Changes in IHTG, liver stiffness, body weight, ALT, glucose, lipids, and APRI	Lifestyle intervention is effective in treating NAFLD in both nonobese and obese patients. Weight reduction predicts remission of NAFLD in nonobese patients, but a modest weight reduction may be sufficient in this population
Xin et al. [108]	Longitudinal cohort study	China	97,699 cancer cases and 304,736 controls of European ancestry	NA	Genetic instrumental variables	Specific to cancer GWAS, we found that circulating vitamin E was significantly associated with increased bladder cancer risk (OR_IVW_ = 6.23, P_IVW_ = 3.05 × 10^-3^) but decreased breast cancer risk (OR_IVW_ = 0.68, P_IVW_ = 8.19 × 10^-3^); however, the significance of breast cancer was dampened (P_multivariable IVW_ > 0.05) in the subsequent multivariable MR analysis. In the validation stage of the UK Biobank cohort, we did not replicate convincing causal effects of genetically predicted circulating vitamin E concentrations and dietary vitamin E intake on the risk of ten cancers
Younes and Bugianesi [14]	Review of literature	Italy	Lean NAFLD patients	NA	NA	Compared with healthy individuals, lean subjects with NAFLD present metabolic risk factors (dyslipidemia, arterial hypertension, diabetes, and insulin resistance) to a significantly greater extent, probably due to a more dysfunctional adipose tissue, not limited to its visceral component. General recommendations include the adoption of a healthy lifestyle
	Multicenter, randomized, placebo-controlled phase 3 trial	United States	332 centers in 20 countries across the world	Histological	Fibrosis improvement (≥1 stage) with no worsening of NASH, or NASH resolution with no worsening of fibrosis	Obeticholic acid 25 mg significantly improved fibrosis and key components of NASH disease activity among patients with NASH. The beneficial effects of obeticholic acid on fibrosis and key components of NASH disease activity were robust, based on the observed consistency of results across multiple histological endpoints with reproducible response ratios, as well as the evident dose-response and markedly consistent benefit across analysis populations. Treatment with obeticholic acid had a beneficial effect on other markers of hepatocellular injury (ALT and AST) and oxidative stress (GGT). Obeticholic acid was generally well tolerated, with a profile that is generally consistent with prior studies
Zelber-Sagi et al. [110]	Prospective study	Italy	799 participants, a subgroup of Israeli adults (aged 24-70 years)	Abdominal US and liver steatosis was quantified noninvasively by HRI and SteatoTest (BioPredictive, France)	Fibrosis progression	High coffee consumption was associated with a lower proportion of clinically significant fibrosis ≥ F2 (8.8% vs. 16.3%; p = 0.038); consistently, in multivariate logistic regression analysis, high coffee consumption was associated with lower odds for significant fibrosis (OR, 5.0.49; 95% CI, 0.25-0.97; p = 0.041) and was the strongest predictor for significant fibrosis

A balanced diet rich in omega-3 fatty acids, fiber, and antioxidants has shown benefits in the management of MASLD [92]. The Mediterranean diet, noted for low calories and increased omega-3 fatty acids, has been associated with improvement in hepatic steatosis, fibrosis, and cardiovascular comorbidities [85,92,95]. A systematic review and meta-analysis by Del Bo' et al. affirmed the role of the Mediterranean diet in decreasing total cholesterol and liver fibrosis, which is the primal determinant of long-term outcomes in MASLD [73]. Furthermore, the literature highlights that lycopene, a soluble pigment found in fruit and vegetables, can be effective against MASLD [112]. Excessive dietary fructose is a known risk factor for MASLD, and reduced consumption of fructose-sweetened products is beneficial [64,66]. Coffee consumption has been associated with a reduced risk of MASLD and a significant decline in fibrosis progression in population-based studies and meta-analyses [76,106,110].

Oxidative stress plays a role in MASLD disease progression; however, caution is recommended with unapproved dietary and herbal supplements marketed for their antioxidant activities [92]. The FDA has approved no drugs for the treatment of MASLD. However, medications including vitamin E, pioglitazone, glucagon-like peptide-1 receptor agonists (RAs), and sodium-glucose cotransporter-2 inhibitors have shown some benefit [109]. Vitamin E as an antioxidant has shown improvement in liver function and histology in MASLD [100]. The Pioglitazone versus Vitamin E versus Placebo for the Treatment of Nondiabetic Patients with Nonalcoholic Steatohepatitis (PIVENS) trial showed benefits in adults with NASH but not type 2 diabetes mellitus treated with pioglitazone (30 mg daily) or vitamin E (800 international units daily) compared with placebo in terms of liver chemistries, hepatic steatosis, and lobular inflammation [96]. There has been conflicting evidence about the role of vitamin E in improving fibrosis in MASLD [78,90,91]. However, its association with a reduction in hepatic decompensation and improved transplant-free survival was evidenced in a retrospective study involving patients with NASH and advanced fibrosis [104]. Potential risks, including hemorrhagic stroke, an increase in all-cause mortality, and conflicting evidence regarding vitamin E and prostate cancer, should be included in treatment options discussions [94,98,113]. Other studies have proven the benefits of pioglitazone in improving liver chemistries and steatosis, insulin resistance, fibrosis, and progression to cirrhosis and HCC in MASLD patients with or without type 2 diabetes mellitus [70,72,74,87]. However, a cross-sectional analysis by Le et al. found a downward trend in pioglitazone utilization in United States adults with type 2 diabetes mellitus and MASLD between 2003 and 2016 despite an increase in this patient population [86]. This might be attributed to the adverse effects of pioglitazone, including weight gain, fractures, and heart failure exacerbation [105]. The 2023 AGA guidelines on the management of lean MASLD reserves the use of Vitamin E or pioglitazone only for biopsy-proven NASH [10].

Statins are safe for patients with elevated cholesterol and MASLD, including compensated cirrhosis, with potential benefits on liver enzymes and cardiovascular risk [94]. A study utilizing the national database of the compulsory national health insurance scheme in South Korea was notable for a reduced risk of MASLD development in patients on statins, with an adjusted odds ratio (AOR) of 0.66 and a 95% CI of 0.65-0.67. The risk of progression to liver fibrosis once MASLD developed was significantly reduced in patients on statins (AOR, 0.43; 95% CI, 0.42-0.44). Both observations were independent of type 2 diabetes mellitus. Fatty liver index and BARD score were used in MASLD and advanced hepatic fibrosis case identification, respectively [28].

Regular monitoring for disease progression and complications: Periodic assessment of liver enzymes, fibrosis biomarkers, and imaging studies to monitor disease progression and efficacy of interventions is essential [103]. These tests and noninvasive fibrosis scoring systems can be monitored every six months to two years, depending on the fibrosis stage and response to therapy [10]. Cirrhosis is a known risk factor for HCC, irrespective of its underlying etiology [65]. The combination of at least two noninvasive tests interpreted per the patients' clinical context is recommended for advanced fibrosis/cirrhosis monitoring [88]. A reduction of 2 or more points in NAS scores and the prevention of fibrosis progression are common criteria of histological response in NASH despite variations across clinical trials [103]. Some studies have shown that patients with lean MASLD have an increased risk of cancer, including gastrointestinal malignancy, compared to nonlean MASLD patients [71,100]. Patients with lean MASLD complicated by cirrhosis should undergo HCC screening with abdominal US with or without serum alpha-fetoprotein [10,65].

Lean MASLD is associated with an increased risk of cardiovascular diseases [83,98]. Patients should undergo early and regular cardiovascular disease screening irrespective of disease/fibrosis stage [82]. A single-center study including 5,907 adults undergoing screening colonoscopy categorized into two cohorts based on US (cohort 1) and transient elastography (cohort 2) as diagnostic tools for NAFLD was significant for an increased risk of developing clinical cardiovascular disease in 10 years in patients with lean NAFLD compared to lean healthy patients (Framingham risk score, regression coefficient cohort I: 1.93, 95% CI = 0.95-2.92, p < 0.003; cohort II: 1.09, 95% CI: 0.81-2.10, p = 0.034) [98]. A systematic review and meta-analysis by Bisaccia et al., including 33 studies with 10 ± 6 years of mean follow-up, found that patients with lean NAFLD have a comparable risk for developing cardiovascular disease and an increased risk of dying (OR, 1.50; 95% CI, 1.12-2.00; p = 0.006) from cardiovascular causes compared with nonlean NAFLD patients [69].

Emerging therapies: Several drugs are under investigation for MASLD, including OCA, elafibranor, selonsertib, VK2809, resmetirom, firsocostat, and aramchol, among many others [77]. OCA, a farnesoid X receptor agonist, remains the most advanced in this group of investigational drugs, showing consistent promise in MASLD-associated fibrosis regression in phase 3 trials [105]. The Randomized Global Phase 3 Study to Evaluate the Impact on NASH with Fibrosis of Obeticholic Acid Treatment (REGENERATE) trial involving 1,968 patients with NASH randomized to placebo, OCA 10 mg, or OCA 25 mg daily demonstrated significant improvement in fibrosis by one stage or more in 23% in the OCA 25 mg group compared with 12% in the placebo group [109]. Despite NASH resolution not achieving statistical significance in all treatment arms, there was an increased improvement in NASH histological profile in the OCA 25 mg group [109]. Pruritus was a common adverse effect of OCA, occurring in more than half of the 25 mg group and raising concerns for compliance [109]. Cardiovascular events were similar across all treatment groups. However, it is important to emphasize that statin use was about fivefold greater (placebo: 66; OCA 10 mg: 155; OCA 25 mg: 159) in the OCA group compared to placebo [109], highlighting the need for heightened cardiovascular surveillance with future OCA use [105].

Resmetirom and VK2809 are liver-specific thyroid hormone receptor alpha and beta agonists with regulatory properties related to lipid metabolism [93]. The resmetirom multicenter phase 2B study demonstrated a relative reduction in hepatic steatosis at week 12 (-32.9% resmetirom vs. -10.4% placebo; p < 0.0001) and 36 (-37.3% resmetirom vs. -8.5% placebo; p < 0.0001) in patients with NASH evaluated by MRI-PDFF [93]. NASH resolution was evaluated with biopsy, and NAS scores were higher in the resmetirom group (27% vs. 6%, p = 0.018) at 36 weeks [93]. Resmetirom was also associated with an improved lipid profile compared to placebo [96]. Similar mild-to-moderate adverse events were recorded in both groups, with the exception of increased transient nausea and diarrhea in the resmetirom group [96].

Challenges in managing lean MASLD: MASLD has traditionally been associated with obese or overweight patients. The lack of awareness of the unique features of the disease in persons with normal body weight can pose an increased risk through a decreased index of suspicion, leading to missed or delayed diagnosis [75,89]. Lean MASLD can have a wide spectrum of presentations, from mild to significant liver inflammation with its attendant complications despite a lean phenotype [14]. This, together with the lack of established guidelines from major societies tailored to the lean MASLD population, given its recent recognition and paucity of data, poses major challenges in patient management.

Discussion

This systematic review aims to evaluate the diagnosis and management of Lean MASLD comprehensively. Characterized by unique features, lean MASLD poses diagnostic and management challenges distinct from those observed in obese counterparts. This work contributes to the existing literature by providing a detailed overview of lean MASLD, offering valuable insights into its diagnosis and management.

Diagnostic criteria prioritize cardiometabolic risk factors over BMI, influencing the prevalence and clinical presentation, often asymptomatic and incidentally diagnosed. Unique features include distinctive clinical and metabolic profiles. Challenges in marker reliability and imaging modalities are discussed, focusing on MRI's potential. Beyond diagnosis, management involves tailored strategies considering the unique metabolic characteristics of lean MASLD, emphasizing the need for vigilant surveillance.

Diagnostic Challenges and Approaches

Diagnosing lean MASLD presents unique challenges that necessitate a nuanced approach. Given the distinct pathophysiology of this condition, emphasizing the pivotal role of cardiometabolic risk factors over BMI alone is crucial. Unlike traditional associations with obesity, lean MASLD demands a heightened awareness of its atypical presentation and the importance of considering comprehensive risk factors for accurate diagnosis.

The limitations and reliability of conventional diagnostic markers, such as ALT and AST, in lean MASLD, underscore the need for a more comprehensive diagnostic strategy. Traditional imaging modalities like US, CT, and MRI are explored in this context. While US demonstrates high sensitivity, its efficacy diminishes in the presence of chronic liver disease and fibrosis. CT scan limitations lie in detecting mild hepatic steatosis, making it unsuitable for widespread screening. In contrast, MRI, particularly MR spectroscopy-derived PDFF, emerges as a promising and accurate tool, surpassing traditional histological assessments.

Noninvasive fibrosis scoring systems and elastography are pivotal in assessing liver fibrosis in lean MASLD. Validated systems, including FIB-4, NFS, and AST-to-platelet ratio index, offer reliable insights into fibrosis risks and overall prognosis. While elastography methods, particularly transient elastography and MR elastography, show promise, their application should be mindful of potential limitations in cases of increasing obesity. The combination of fibrosis scoring systems and elastography, either concurrently or sequentially, emerges as a highly accurate diagnostic strategy, offering significant potential for improving diagnostic precision in lean MASLD.

Clinical Characteristics of Lean MASLD

Comparing the clinical characteristics of lean MASLD with other MASLD subtypes unveils distinct features, notably in age, metabolic parameters, and symptomatic presentation. Noteworthy differences include a younger age demographic in lean MASLD patients and a unique metabolic profile marked by lower fasting glucose, HbA1c, blood pressure, and HOMA-IR levels. Symptomatically, lean MASLD tends to be asymptomatic, but presentations may include fatigue, malaise, or mild abdominal discomfort.

The significance of early detection in lean MASLD cannot be overstated. Unlike its obese counterparts, the lean phenotype is associated with lower cardiovascular risk factors, underscoring the importance of timely identification. Early detection not only facilitates appropriate management but also provides an opportunity to mitigate the risk of cardiovascular morbidity and mortality. The unique association between lean MASLD and a reduced cardiovascular risk profile highlights the need for tailored diagnostic strategies that consider both hepatic and cardiovascular health.

Understanding the clinical nuances of lean MASLD contributes to a proactive approach to patient care. Recognizing the age-related differences, metabolic distinctions, and often-subtle symptomatic presentations allows for targeted diagnostic efforts, emphasizing the need for vigilance in the absence of traditional risk factors associated with obesity. This heightened awareness enables healthcare providers to intervene early, optimize management strategies, and mitigate the long-term impact on both hepatic and cardiovascular outcomes.

Importance of Liver Fibrosis Assessment

Early detection of liver fibrosis in lean MASLD holds critical importance as a predictor of disease progression, cirrhosis, and adverse outcomes. Timely identification enables proactive intervention, potentially mitigating the long-term impact on liver health and associated complications. Studies, such as the meta-analysis by Sookoian and Pirola, highlight a substantial increment in mean fibrosis in overweight/obese subjects compared to lean/nonobese MASLD counterparts, underscoring the value of fibrosis assessment in predicting disease severity and progression [61].

Noninvasive fibrosis scoring systems play a pivotal role in this context, providing a valid and accurate means of assessing fibrosis in lean MASLD. Validated systems such as FIB-4, NFS, and AST-to-platelet ratio index offer reliable insights into fibrosis risks, with elevated scores associated with increased risks of adverse liver events and mortality [40,54]. The enhanced accuracy of serological tests like ELF and Hepascore, which include direct fibrosis biomarkers, further underscores their potential in predicting advanced fibrosis [29]. Although not specifically validated in lean/nonobese MASLD patients, studies suggest comparable or even higher performance at detecting advanced fibrosis in lean compared to obese MASLD [36,37].

Elastography, particularly transient elastography and MR elastography, emerges as an accurate tool for assessing liver fibrosis in lean MASLD. Their superiority over traditional noninvasive fibrosis tests and scoring systems is notable, with studies showcasing promising results. The combination of fibrosis scoring systems and elastography, either concurrently or sequentially, demonstrates high accuracy, offering a potential breakthrough in assessing fibrosis, disease progression, and mortality risk in lean MASLD [30,31,47,62]. The study by Boursier et al., utilizing a sequential combination of FIB-4 followed by FibroMeter-VCTE, demonstrated a high accuracy of 90% in diagnosing advanced fibrosis, with biopsy required in only 20% for confirmation [30].

Management Strategies

General management for lean MASLD involves lifestyle modifications, focusing on diet and increased physical activity. The cornerstone of intervention is weight loss, aiming for 3%-5%, with evidence supporting its efficacy in improving hepatic steatosis, NAS scores, and histological profiles [67,68,79]. However, sustaining weight loss poses challenges, necessitating a multidisciplinary approach involving nutrition services, psychological support, and social systems [94,101,102]. Studies, like the trial from China and prospective studies from Bangladesh, demonstrate the positive impact of weight reduction on hepatic steatosis, NAS scores, and histology, with greater gains maintained by nonobese groups [27,107].

A balanced diet rich in omega-3 fatty acids, fiber, and antioxidants holds promise for managing lean MASLD. The Mediterranean diet, recognized for its benefits in hepatic steatosis, fibrosis, and cardiovascular comorbidities, is particularly highlighted [85,92,98]. Evidence from systematic reviews and meta-analyses, such as the study by Del Bo' et al., further supports the role of the Mediterranean diet in decreasing total cholesterol and liver fibrosis [73]. Reducing excessive dietary fructose and embracing coffee consumption are additional dietary considerations with potential benefits [64,66,76,106,110].

While no FDA-approved drugs exist for lean MASLD, vitamin E and pioglitazone show some promise. The PIVENS trial and retrospective studies indicate potential benefits in improving liver chemistries, steatosis, and inflammation [70,72,74,87,96,104]. However, the use of vitamin E, despite its potential benefits, is nuanced due to conflicting evidence on fibrosis improvement and associated risks [78,90,91,104,108,113]. Statins, deemed safe for elevated cholesterol in MASLD, also demonstrate potential benefits in reducing MASLD development and progression to liver fibrosis [28]. The evidence suggests a need for careful consideration and patient-specific approaches, with guidelines recommending vitamin E or pioglitazone only for biopsy-proven NASH [10].

Monitoring and Surveillance

Regular monitoring for disease progression in lean MASLD is imperative, employing liver enzymes, fibrosis biomarkers, and imaging studies. Periodic assessments, every six months to two years, facilitate timely intervention and enhance the understanding of disease dynamics. The increased risk of HCC in cirrhotic patients underscores the necessity of HCC screening with abdominal US and serum alpha-fetoprotein. Furthermore, the association between lean MASLD and an elevated risk of cardiovascular diseases emphasizes the importance of early and regular cardiovascular screening irrespective of disease or fibrosis stage [65,98]. Vigilant monitoring allows for informed decision-making, optimizing patient care and reducing the risk of complications.

Emerging Therapies and Future Directions

Current investigations into MASLD drug therapies, notably OCA, resmetirom, and VK2809, show promising developments. The REGENERATE trial highlights OCA's potential in fibrosis regression, yet challenges such as pruritus and cardiovascular surveillance underscore the need for cautious implementation [105,109]. Resmetirom and VK2809, liver-specific thyroid hormone RAs, demonstrate favorable outcomes in hepatic steatosis reduction and lipid profile improvement, presenting potential breakthroughs [81,93].

Future directions in lean MASLD research encompass a comprehensive approach to refine diagnostics, tailor therapeutic strategies, and enhance our understanding of the condition. Ongoing investigations should seek to refine diagnostic criteria, exploring novel biomarkers and imaging modalities that improve accuracy and reliability in detecting lean MASLD. Tailoring therapeutic approaches to the unique metabolic profile of lean MASLD is a priority, necessitating research on the effectiveness and safety of lifestyle modifications, dietary interventions, and potential medications in diverse patient populations. Comprehensive fibrosis assessment tools, specifically validated for lean MASLD, should be a focus, integrating them into routine clinical practice for robust risk stratification.

Longitudinal studies are crucial to assess the long-term efficacy and safety of emerging therapies like OCA, resmetirom, and VK2809 in lean MASLD patients, providing insights into fibrosis regression, sustained improvement, and potential adverse effects. Given the increased cardiovascular risk associated with lean MASLD, future research should develop and validate comprehensive cardiovascular risk assessment tools tailored to this patient population, elucidating the interplay between hepatic and cardiovascular outcomes. Investigating the effectiveness of multidisciplinary care models, patient-reported outcomes, and health economic assessments will further enrich our understanding of lean MASLD and guide holistic patient management, ensuring optimal outcomes and resource allocation. Future research endeavors should adopt a multidimensional and patient-centered approach to advance our knowledge and care practices in lean MASLD.

Strengths and Limitations of the Study

This systematic review of lean MASLD contributes to the existing body of knowledge by comprehensively evaluating its diagnosis and management. Several strengths characterize this study, enhancing its credibility and utility. The review provides a thorough and up-to-date synthesis of current literature on lean MASLD, offering a comprehensive overview of diagnostic criteria, clinical characteristics, management strategies, and emerging therapies. By delving into diagnostic challenges, clinical features, and management approaches, the study adopts a multidimensional narrative analysis, offering insights into the complexity of lean MASLD beyond a singular focus. Including recent findings from various studies and consensus statements ensures the incorporation of the latest developments in the field, enhancing the relevance and timeliness of the review.

Despite these strengths, certain limitations should be acknowledged. The review relies on the available literature, and variations in study methodologies, populations, and diagnostic criteria across different research may introduce inherent biases. Additionally, the rapidly evolving nature of MASLD research means that some of the information presented may become outdated over time. While efforts were made to encompass a diverse range of sources, the potential for publication bias and the exclusion of non-English language studies may limit the comprehensiveness of the review.

Future Research

Future research on lean MASLD should focus on refining diagnostic criteria with novel biomarkers and advanced imaging modalities to improve accuracy. Tailoring therapeutic strategies to the unique metabolic profiles of lean MASLD patients is essential, including studies on the efficacy and safety of lifestyle modifications, dietary interventions, and potential medications. Developing and validating specific fibrosis assessment tools for lean MASLD will aid in robust risk stratification. Longitudinal studies on emerging therapies like OCA, resmetirom, and VK2809 are needed to assess their long-term impact on disease progression. Additionally, research should address the cardiovascular risks in lean MASLD by creating specialized risk assessment tools. Exploring multidisciplinary care models and patient-reported outcomes will further enhance our understanding and management of lean MASLD.

## Conclusions

Lean MASLD stands out as a distinct and unique clinical entity, warranting vigilant attention for timely diagnosis and appropriate management. Clinicians must recognize its unique characteristics and potential implications to provide optimal care. Patient education is pivotal, emphasizing the significance of regular monitoring and adherence to therapeutic recommendations for effective disease management.

While this review provides valuable insights into the current understanding of lean MASLD, the evolving nature of research in this field necessitates continued investigation. Future studies should focus on refining diagnostic criteria, unraveling the nuanced clinical features, and developing targeted treatments tailored to the specific needs of this patient population. By advancing our knowledge and refining clinical approaches, ongoing research will contribute to improving the outcomes and quality of life for individuals with lean MASLD.

## Appendices

Appendix 1

**Table 5:** Literature search keywords BMI: body mass index; MASLD: metabolic dysfunction-associated steatotic liver disease; MeSH: Medical Subject Headings; NAFLD: nonalcoholic fatty liver disease

S. no.	Journal databases keyword strategy search keywords
1	Outcome of interest: MASLD ("Lean Metabolic Dysfunction-Associated Steatotic Liver Disease" [Title/Abstract] OR "fatty liver" [Title/Abstract] OR "hepatic steatosis" [Title/Abstract] OR " MASLD" [Title/Abstract])
2	Population of interest: lean individuals ("lean" [Title/Abstract] OR "non-obese" [Title/Abstract] OR "normal weight" [Title/Abstract] OR "low BMI" [Title/Abstract]) AND ("Non-alcoholic Fatty Liver Disease" [MeSH] OR "fatty liver" [Title/Abstract] OR "hepatic steatosis" [Title/Abstract] OR "NAFLD" [Title/Abstract])
3	Diagnosis and management ("Clinical Presentation"[Title/Abstract] OR "Liver Enzymes"[Title/Abstract] OR "Imaging"[Title/Abstract] OR "Liver Biopsy"[Title/Abstract] OR "Lifestyle Modifications"[Title/Abstract] OR "Diet"[Title/Abstract] OR "Physical Activity"[Title/Abstract] OR "Pharmacotherapy"[Title/Abstract] OR "Statins"[Title/Abstract] OR "Regular Monitoring"[Title/Abstract] OR "Management of Complications"[Title/Abstract] OR "Emerging Therapies"[Title/Abstract] OR "Challenges in Managing Lean MASLD"[Title/Abstract] OR "Lack of Awareness"[Title/Abstract] OR "Variable Presentation"[Title/Abstract] OR "Unestablished Treatment Guidelines"[Title/Abstract] OR "Timely Diagnosis"[Title/Abstract] OR "Appropriate Management"[Title/Abstract] OR "Research on Lean MASLD"[Title/Abstract] OR "Research on Lean MASLD"[Title/Abstract] OR "Targeted Treatments"[Title/Abstract])

Appendix 2

**Table 6:** Eligibility criteria for studies upon screening the search results obtained MASLD: metabolic dysfunction-associated steatotic liver disease; NPV: negative predictive value; PPV: positive predictive value; RCT: randomized controlled trial

Criteria	Inclusion	Exclusion
Study design	RCTs, observational studies (cohort, case-control, cross-sectional), literature reviews, systematic reviews, and meta-analyses	Case reports, case series, editorials, animal studies
Participants	Human participants diagnosed with lean MASLD	Studies not directly related to MASLD or involving populations outside the scope of lean individuals with metabolic dysfunction-associated steatotic liver disease
Interventions/exposures	Various interventions related to the diagnosis and management of MASLD, including diagnostic modalities (clinical, imaging, and biopsy), lifestyle modifications, pharmacotherapy, and emerging therapies	Interventions or exposures not directly related to the diagnosis or management of MASLD
Outcomes	Diagnosis: diagnostic accuracy (sensitivity, specificity, PPV, and NPV) and clinical characteristics. Management: therapeutic outcomes, improvements in liver function, reduction in hepatic steatosis, resolution of metabolic dysfunction, and adverse events	Nonrelevant outcomes or studies lacking relevant outcome data
Language	Studies published in English language	Studies published in languages other than English
Population	No restrictions based on demographics or clinical characteristics	Studies focusing on populations not relevant to lean MASLD
Study type	Studies explicitly addressing lean MASLD, its diagnosis, and management	Studies not directly related to lean MASLD
